# Efficacy and immunomodulatory effect of Claudin18.2-specific IL-7/XCL1 armored CAR-T cells in digestive tract cancer: preclinical and clinical analysis

**DOI:** 10.1038/s41392-026-02621-8

**Published:** 2026-03-09

**Authors:** Xuan Zhao, Jinyan Liu, Zhen Zhang, Yali Zhou, Shuiling Jin, Hong Zong, Feng Wang, Min Song, Yali Zhong, Qinglong Li, Bo Pei, Yong Yu, Ming Gao, Wengang Ge, Lu Han, Jiangtao Ren, Yi Zhang

**Affiliations:** 1 Biotherapy Center & Cancer Center, The First Affiliated Hospital of Zhengzhou University, Zhengzhou, China; 2 Nanjing Bioheng Biotech Co., Ltd, Nanjing, China; 3 Department of Oncology, The First Affiliated Hospital of Zhengzhou University, Zhengzhou, China; 4 Department of Radiology, The First Affiliated Hospital of Zhengzhou University, Zhengzhou, China; 5 School of Life Sciences, Zhengzhou University, Zhengzhou, Henan China; 6 School of Public Health, Zhengzhou University, Zhengzhou, Henan China; 7 Tianjian Laboratory of Advanced Biomedical Sciences, Academy of Medical Sciences, Zhengzhou University, Zhengzhou, Henan China

**Keywords:** Tumour immunology, Immunotherapy

## Abstract

Chimeric antigen receptor (CAR)-T cell therapy exerts limited therapeutic efficacy in solid tumors including digestive tract cancer (DTC), which is largely attributable to the suppressive tumor microenvironment (TME) and the functional deficits of CAR-T cells. Herein, we generated fourth-generation CAR-T cells engineered to target Claudin18.2 (CLDN18.2) with concurrent secretion of IL-7 and XCL1, which are designated as ExCAR-T cells (also named RD07 cells in a clinical trial). The preclinical results demonstrated the remarkable and enduring suppressive effects of ExCAR-T cells on DTC growth in murine models through activating both the inherent of the administered CAR-T cells and robust endogenous immune cells anti-tumor response. Furthermore, we performed a clinical investigation for previous systemic treatment failed patients with DTC. RD07 therapy was well tolerated, and 7 out of 10 patients exhibited tumor regression; this effect was particularly evident in patients exhibiting moderate to high CLDN18.2 expression (DCR of 100%). Finally, single-cell RNA (scRNA) sequencing combined with spatial landscape profiling revealed that RD07 has antitumor effects and activates endogenous immune cells within the TME. Concomitantly, enhanced cytotoxic activity of CAR-T cells and expanded T cell receptor (TCR) clonotypes were detected in patients with a partial response (PR). Taken together, present data demonstrate the therapeutic efficacy and safety of RD07 in our study and highlight its ability to both exert antitumor effects and remodel the TME. These findings support RD07 as an innovative CAR-T cell therapy for DTC.

## Introduction

Cancer still ranks as the most common cause of death worldwide. Traditional therapeutic modalities, encompassing surgical intervention, radiotherapy, chemotherapy and targeted therapy, have been recognized as the standard treatments and indeed reduced the death rates in the past decades. However, the clinical response is still far from satisfactory, and innovative treatments are needed to resolve this clinical dilemma. Chimeric antigen receptor (CAR)-T cell therapy was engineered to specifically eliminate tumor cells through transforming a scFv that binding to tumor cells surface antigen to T cells.^1^ Plenty researches have convinced the superior anti-tumor ability of CAR-T cells in basic research. This encouraged researchers to perform clinical trials to assess the safety and therapeutic efficacy of CAR-T cell therapy. Consistent with basic research, CAR-T cell therapy has demonstrated striking clinical efficacy in the treatment of advanced hematopoietic malignancies, with an ORR reaching up to 90%,^2,3^ which has led to the approval of 13 CAR-T cell therapies across the globe. However, solid tumors account for close to 90% of all malignant neoplasms with disappointing clinical response, the innovative treatment option is urgently needed. Motivated by the remarkable clinical efficacy of CAR-T cell therapy in hematologic malignancies, researchers performed clinical trials for solid tumor treatment. Heczey A and colleagues reported the clinical application of GPC3-targeted CAR-T cell therapy in patients with solid tumors, which achieved a 66% disease control rate and a 33% antitumor response rate.^4^ In a separate clinical trial, CAR-T cells engineered to target IL-13Rα2 yielded a 50% rate of stable disease or better responses in patients with recurrent glioblastoma (rGBM).^5^ Though the therapies of CAR-T cell have exhibited prospective clinical safety and efficacy against hematopoietic malignancies, they have thus far been less efficacious in the management of solid malignancies.^6,7^

Digestive tract cancers (DTCs) account for over a quarter of all cancer cases globally and are responsible for one-third of cancer-associated mortalities. Among cancers of the digestive tract, gastric cancer (GC) and pancreatic cancer (PC) are among the deadliest; these cancers respond poorly to current therapies, such as maximal surgical resection, chemotherapy and focal radiotherapy.^8^ Therefore, CAR-T cell therapy is currently under active investigation for its ability to improve cancer treatment outcomes.^9^ Although multiple CAR-T cell therapies, among which targeting MUC1,^10^ MLSN,^11^ CEA, HER2,^12^ PSCA,^13^ CLDN18.2^7^ and CD133,^14^ have shown feasibility and safety for PC and GC treatment in preclinical and clinical studies, their efficacy remains insufficient. Choosing a specific tumor antigen is an important considers for CAR-T cells production. CLDN family, functioning as tetraspan-transmembrane proteins at tight junctions, has been identified as a promising therapeutic target due to its specific expression in tumor cells. And CLDN6 and CLDN18.2 CAR-T cells were widely accepted as ideal targets for the specifically upregulation in human solid tumor cells. CLDN6-directed CAR-T cells exhibited superior clinical efficacy in advanced solid tumor with ORR and DCR reaching 33% and 67%, respectively in a clinical trial.^15^ Lin Shen and her colleagues reported that CLDN18.2 as a promising therapeutic target for gastric cancer.^16,17^ In this study, we selected CLDN18.2 as the therapeutic target for CAR-T cell therapy in DTC.

After specifying the target, we cannot ignore the suppressive tumor microenvironment (TME), intratumoral heterogeneity, and insufficient expansion and impaired persistence of CAR-T cells in DTC.^18^ To surmount these hurdles within the microenvironment of solid tumors, researchers have explored various approaches for engineering CAR-T cells.^19^ Thus, in this study, we intended to generate a fourth generation CLDN18.2-targetd CAR-T cells and aimed to achieve better anti-tumor response for DTC patients. Given the critical role of crosstalk between conventional type 1 dendritic cells (cDC1s) and T cells in driving robust antitumor immune responses within solid tumors,^20^ boosting the crosstalk between cDC1s and T cells is one approach that is considered promising for promoting tumor eradication. In this context, many innovative CAR designs that incorporate cytokines and chemokines to improve CAR-T cell expansion and infiltration and promote systemic immune surveillance by recruiting dendritic cells have been developed.^6,21^ However, none have been tested in the clinic.

It is well established that IL-7 exerts an irreplaceable role in the survival and homeostasis of T cells, and XCL1 is a major chemokine for cDC1s that confers on them critical functions in orchestrating the establishment of the T-cell zone and stimulating endogenous T-cell responses by promoting systemic immune activation^22,23^; thus we constructed CAR-T cells with the capacity to secrete IL-7 and XCL1, which was designed to enhance the infiltration and accumulation of cDC1s and T cells within the TME. Specifically, we performed genetic modification on CAR-T cells to enable the targeted recognition of CLDN18.2, an antigen validated as an exceptionally promising therapeutic target in gastrointestinal malignancies. We engineered these cells to concurrently secrete IL-7 and XCL1, which we term “Explore CAR-T” (ExCAR-T) cells; preclinical investigations confirmed their safety and therapeutic efficacy against gastrointestinal malignancies. Furthermore, we conducted a single-arm, open-label, dose-escalation trial to evaluate the safety and therapeutic efficacy of RD07 (ExCAR-T cells) in patients with advanced digestive tract cancer. Finally, the results of the single-cell RNA sequencing (scRNA-seq) and imaging mass cytometry (IMC) analyses confirmed that RD07 reprograms the TME by activating endogenous T cells and facilitating their interaction with cDC1s. This approach shows great potential to enhance the therapeutic efficacy of CAR-T cells via the induction of an endogenous antitumor immune response.

## Results

### ExCAR-T cells have greater enhanced antitumor capacity

**Fig. 1:**
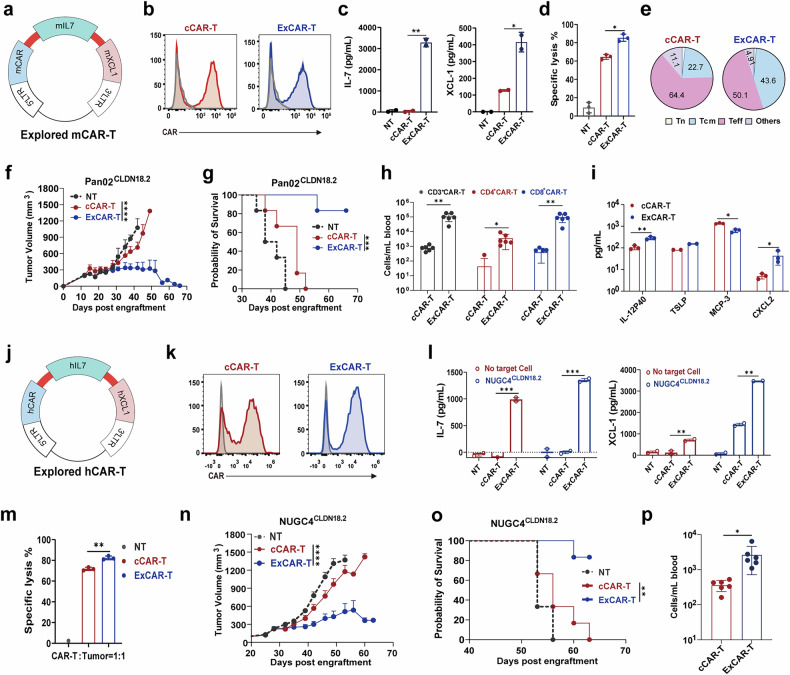
Compared with conventional CLDN18.2 CAR-T cells, IL-7/XCL1 armored CAR-T cells exhibit greater enhanced antitumor capacity. **a** Schematic of the murine ExCAR construct. **b** Staining of the CAR on murine T cells 5 days after retroviral CAR transduction. **c** Concentrations of IL-7 and XCL1 in the day 4 to day 5 CAR-T cell supernatants were examined via ELISA (*n* = 2). **d** Cytotoxicity assay of anti-murine CLDN18.2 cCAR-T and ExCAR-T cells with Pan02^CLDN18.2^ cells as targets (*n* = 3). The effector/target ratio was 1:1. **e** Murine CAR-T cells cultured for 5 days were defined as Tn, Tcm, Teff, or other cells on the basis of staining for CD44 and CD62L (*n* = 1). **f** Tumor volume in the different treatment groups. C57BL/6 mice were inoculated subcutaneously (s.c.) with Pan02^CLDN18.2^ cells on day 0, treated with cyclophosphamide (CPA) on day 9, and administered anti-CLDN18.2 CAR-T cells on day 12 (*n* = 6). **g** Kaplan‒Meier survival curves for each treatment group; statistical analysis was performed via the log-rank test (*n* = 6). **h** CAR-T cell counts detected by flow cytometry in the Pan02^CLDN18.2^ mouse model on day 21 after CAR-T cell therapy (*n* = 6). **i** IL12‒P40, TSLP, MCP-3, and CXCL2 levels in the serum of the mice in the different treatment groups (*n* = 3). **j** Schematic of the human ExCAR construct. **k** CAR expression in human CAR-T cells 9 days after transduction with retrovirus. **l** IL-7 and XCL1 secretion by CAR-T cells in the absence or presence of NUGC4^CLDN18.2^ cells. Target cells (2 × 10^5^/well) were mixed with anti-hCLDN18.2 cCAR-T cells or ExCAR-T cells (2 × 10^5^/well). The concentrations of IL-7 and XCL1 in the supernatants after 16 hours of coculture were measured via ELISA (*n* = 2). **m** Cytotoxicity assay of anti-hCLDN18.2 cCAR-T or ExCAR-T cells in which NUGC4^CLDN18.2^ cells were used as targets (*n* = 3). **n** Tumor volume in the different treatment groups. NPI mice were subcutaneously inoculated with NUGC4^CLDN18.2^ cells on D0 and treated with antiCLDN18.2 CAR-T cells 25 days later (*n* = 6). **o** Kaplan‒Meier survival curves for each treatment group; statistical analysis was performed via the log-rank test (*n* = 6). **p** CAR-T cell count detected by flow cytometry in the NUGC4^CLDN18.2^ model on day 14 after CAR-T cell treatment (*n* = 6). *P* values from **c**, **d**, **h**, **i**, **l**, **m**, **p** were determined via two-sided unpaired *t* tests. *P* values from **f** and **n** were calculated via two-way ANOVA. The data are presented as the mean ± SEM. ****P < 0.0001; ***P < 0.0005; **P < 0.001; *P < 0.05

To improve CAR-T cell infiltration, expansion and antigen spreading in digestive tract cancers, we engineered fourth-generation murine CAR-T cells coexpressing a CLDN18.2-specific CAR along with IL-7 and XCL1, termed ExCAR-T cells (Fig. 1a). Second-generation CLDN18.2 CAR-T cells lacking IL-7 and XCL1, referred to as conventional CAR-T (cCAR-T) cells, served as controls. CAR expression levels were comparable between ExCAR-T cells and cCAR-T cells (Fig. 1b). Compared with nontransduced T (NT) cells or cCAR-T cells, ExCAR-T cells secreted significantly more IL-7 and XCL1 (Fig. 1c). In an in vitro cytotoxicity assay, ExCAR-T cells demonstrated superior tumor-killing activity against CLDN18.2-overexpressing Pan02 cells (Fig. 1d). Phenotypic analysis revealed a significantly greater proportion of central memory T (Tcm) cells among ExCAR-T cells than among cCAR-T cells (Fig. 1e), suggesting enhanced stemness and persistence.

Next, we constructed a Claudin18.2-bearing Pan02 (Pan02^CLDN18.2^) syngeneic C57BL/6 mouse model and intravenously injected it with murine cCAR-T, ExCAR-T, or NT cells. Compared with cCAR-T cells, ExCAR-T cells suppressed tumor growth and prolonged survival to a greater extent (Fig. 1f, g). Flow cytometry analysis of CAR-T cells in the peripheral blood on day 21 posttreatment revealed that the number of proliferating CAR-T cells, predominantly CD8^+^ T cells, was significantly greater in the ExCAR-T cell group than in the control group (Fig. 1h). Additionally, the ExCAR-T cell group presented increased levels of IL12-P40, TSLP, and CXCL2, which have been implicated in inflammatory responses and immune cell recruitment^24,25^, but exhibited significantly decreased MCP-3 levels (Fig. 1i), a factor known to promote tumor progression by supporting the TME and facilitating invasion and metastasis.^26^

To further verify the remarkable antitumor response of ExCAR-T cells, we constructed CT26^EGFRVIII^, CT26^CLDN18.2^, and Pan02^CD19^ syngeneic mouse models and observed similar tumor regression (Supplementary Fig. 1a–c), underscoring their potential for eliminating tumors. Notably, within the Pan02^CD19^ model, ExCAR-T cells exhibited more potent tumor suppression and B-cell elimination than 7 × 19 CAR-T cells, a CAR-T engineered to secrete IL-7 and CCL19 that has been documented to enhance dendritic cell infiltration into tumor tissues and trigger antigen spreading (Supplementary Fig. 1d).^22^ In the ExCAR-T cell-treated group, 4 out of the 6 mice achieved complete tumor clearance, whereas in the 7 × 19 CAR-T cell-treated group, only 1 out of the 6 mice achieved complete tumor clearance.

On the basis of these findings, we developed human ExCAR-T cells and assessed their efficacy (Fig. 1j). CAR expression levels were comparable between ExCAR-T cells and cCAR-T cells (Fig. 1k). Compared with cCAR-T cells, ExCAR-T cells exhibited robust IL-7 and XCL1 secretion before and after tumor coculture (Fig. 1l) and increased tumor-killing activity (Fig. 1m). However, this increase in cytotoxicity did not lead to increased IL-2, IFN-γ, or GM-CSF secretion (Supplementary Fig. 1e), reducing the risk of cytokine release syndrome (CRS). Compared with cCAR-T cells, ExCAR-T cells demonstrated superior tumor control and prolonged survival in vivo (Fig. 1n, o). Additionally, a greater proportion of CAR-T cells was detected in the peripheral blood on day 14 after treatment in the ExCAR-T cell group (Fig. 1p). In a patient-derived xenograft (PDX) model, ExCAR-T cells also exhibited superior expansion compared with cCAR-T cells (Supplementary Fig. 1f–i). Collectively, these results demonstrate that ExCAR-T cells effectively control tumor progression and promote expansion in solid tumor models.

### ExCAR-T cells induce systemic immune activation by recruiting cDC1s

**Fig. 2:**
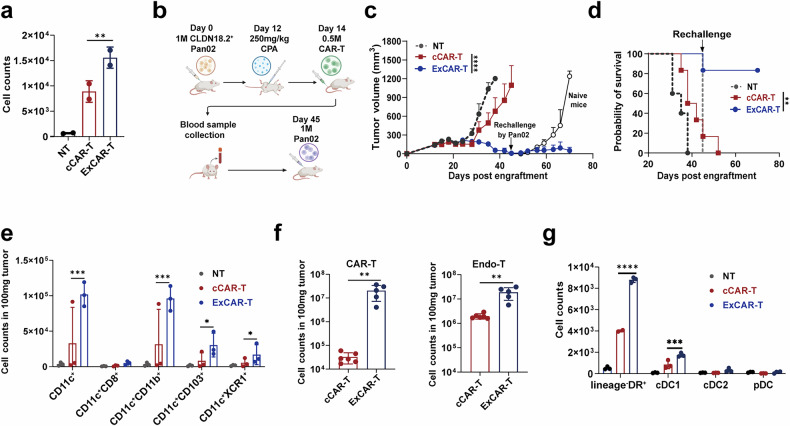
IL-7/XCL1-overexpressing CAR-T cells induce systemic immune activation by recruiting cDC1s into tumors. **a** Chemotaxis of murine responder DCs measured by Transwell assay (8 µm, Corning). Murine CAR-T cell supernatant was added to the lower chambers, and the responder cells (Mutu DC 1940) were incubated in the upper chambers for 18 h. The quantity of cells that transmigrated from the upper chamber to the lower chamber was quantified by flow cytometry (*n* = 2). **b** Schematic chart of the Pan02^CLDN18.2^ mouse model. **c** Tumor volume in the different treatment groups. C57BL/6 mice were inoculated with Pan02^CLDN18.2^ cells and subsequently treated with either anti-hCLDN18.2 cCAR-T cells or ExCAR-T cells. On day 33 after ExCAR-T cell treatment, both the PBS control group and the ExCAR-T treated group were inoculated subcutaneously with Pan02 cells lacking CLDN18.2 expression (*n* = 6). **d** Kaplan‒Meier survival curves for each treatment group; statistical analysis was performed via the log-rank test (*n* = 6). **e** Tumor-infiltrating DC subsets and T cells were detected via flow cytometry on day 28 (*n* = 3). **f** CAR-T and endogenous T (Endo-T) cell numbers in the peripheral blood were detected by flow cytometry on day 49 after CAR-T cell treatment (*n* = 6). **g** Chemotaxis of human responder DCs measured by Transwell assay. Human CAR-T cell supernatant was added to the lower chambers, and the responder cells (Pan DC cells sorted with a Miltenyi Pan DC kit) were incubated in the upper chambers for 18 h (*n* = 3). The quantity of cells that transmigrated from the upper chamber to the lower chamber was quantified by flow cytometry. *P* values from **a** and **e**–**g** were determined via two-sided unpaired *t* tests. *P* values from **c** were determined via two-way ANOVA. The data are presented as the mean ± SEM. ****P < 0.0001; ***P < 0.0005; **P < 0.001; *P < 0.05

To evaluate the ability of ExCAR-T cells to prevent tumor recurrence and circumvent antigen escape mechanisms often driven by antigen downregulation or immune editing, we investigated whether ExCAR-T cells promote tumor control by recruiting cross-presenting cDC1s. To assess the chemotactic effect of XCL1 secreted by ExCAR-T cells, we conducted an in vitro chemotaxis assay using murine cDC1-like Mutu DCs as responder cells. Supernatants from ExCAR-T cells significantly promoted the chemotaxis of Mutu DCs (Fig. 2a).

In a Pan02^CLDN18.2^ tumor model, we rechallenged tumor-free mice from the ExCAR-T group with CLDN18.2-negative Pan02 cells. Remarkably, these mice remained tumor free, suggesting the establishment of long-term immune surveillance mediated by endogenous T cells (Fig. 2b–d). Further analysis revealed an increased proportion of cDC1s in the lymph nodes, spleen, and tumor tissues of ExCAR-T treated mice, indicating increased immune activation (Fig. 2e). Flow cytometry analysis of tumor-infiltrating lymphocytes (TILs) revealed a greater abundance of both endogenous T cells and CAR-T cells in the ExCAR-T cell-treated group (Fig. 2f).

A similar effect was observed in a Pan02^CD19^ model, in which tumor control after rechallenge was achieved in a greater proportion of mice treated with ExCAR-T cells (4/6) than in those treated with 7 × 19 CAR-T cells (1/6) (Supplementary Fig. 2c–e). Additionally, ExCAR-T cells induced greater cDC1 infiltration in the lymph nodes, spleen, and tumor tissues than 7 × 19 CAR-T cells did (Supplementary Fig. 2f). Consistent with these findings, in vitro chemotaxis assays using human pan DCs as responder cells confirmed that human ExCAR-T cell supernatants significantly increased the chemotaxis of human DCs, particularly cDC1s (Fig. 2g, Supplementary Fig. 2b). Together, these data demonstrate that ExCAR-T cells enhance systemic immune activation by recruiting endogenous antigen-presenting cells and activating endogenous T cells, generating an inflammatory TME that effectively suppresses tumor growth, recurrence, and immune evasion.

### Efficacy of RD07 in a clinical trial

For the clinical trial, the ExCAR-T cell product was designated as RD07. To achieve promising preclinical results, we launched a clinical trial to evaluate the safety and therapeutic efficacy of RD07 in patients diagnosed with advanced solid tumors (http://www.chictr.org.cn, ChiCTR2000038650).

By July 1, 2023, 12 patients diagnosed with digestive tract malignancies had been enrolled in the study, among whom 10 presented with gastric cancer or gastroesophageal junction cancer (GC/GEJC) and 2 with pancreatic cancer (PC). All enrolled participants had undergone a minimum of two prior lines of treatment and presented an Eastern Cooperative Oncology Group (ECOG) performance status score of 0–2. The median age of the study population was 56.5 years with an age range spanning from 42 to 70 years, and all patients presented with metastatic disease (Supplementary Table 1). Six patients had metastases in ≥3 organs, and 8 had undergone prior surgical intervention. Medium-to-high expression level of CLDN18.2 (≥40% tumor-positive cells with ≥2+ staining intensity by immunohistochemistry) was observed in eight patients. Half of the cohort (6/12) had an ECOG score of 2 at enrollment. No bridging therapy was conducted. Prior to RD07 infusion (0.1–2 × 10⁷ cells/kg body weight), all patients were administered lymphodepleting chemotherapy consisting of cyclophosphamide, fludarabine, or nab-paclitaxel (Supplementary Table 2). Four patients received multiple infusions.

CRS and immune effector cell-associated neurotoxicity syndrome (ICANS) represent major adverse events (AEs) associated with CAR-T cell treatment. In our trial, over a median follow-up of 203 days (range: 12–466; Fig. 3a), grade 3–4 AEs were predominantly hematologic toxicities attributable to preconditioning (Table 1). Postinfusion cytokine elevation was observed in the peripheral blood (Supplementary Fig. 3c). Eight patients developed grade 1 CRS; no ICANS occurred. Two patients experienced Grade 2 gastrointestinal hemorrhage. No treatment-related deaths were recorded. Patient 5 died on day 12 postinfusion due to pulmonary inflammation and chronic malnutrition. Patient 12 self-administered S-1 (an oral chemotherapeutic agent) from apheresis until 14 days postinfusion, violating the trial protocol. Both patients’ data were included in the reporting of AEs but were excluded from the efficacy analysis.

**Fig. 3:**
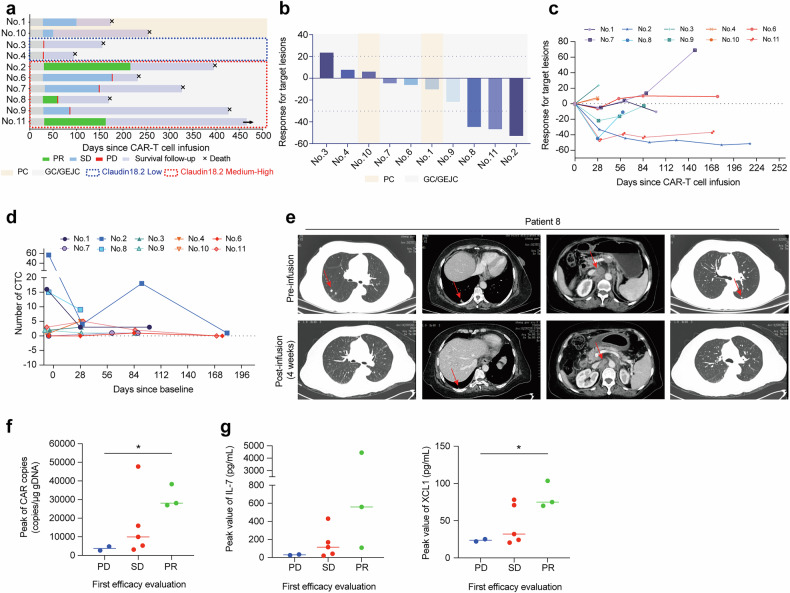
Clinical efficacy after RD07 infusion and analysis of different clinical outcomes. **a** Swimmer plot illustrating the clinical results for each patient. **b** Waterfall plot depicting the best clinical response observed in each patient. **c** Spider plot demonstrating the changes in all target lesions for each patient. **d** Variations in the count of circulating tumor cells (CTCs) within peripheral blood samples following RD07 infusion in individual patients. **e** Computed tomography (CT) scans of metastatic lesions in Patient 8 taken before and four weeks after RD07 infusion. **f**, **g** CAR copy numbers in peripheral blood and the IL7/XCL1 expression in patients with varying responses at the first efficacy evaluation. The data are presented as the mean ± SEM. *P < 0.05 according to one-way ANOVA

**Table 1:** Adverse events occurring in all patients, by worst grade

Adverse event, *n* (%)	Grade 1	Grade 2	Grade 3	Grade 4	Any grade
*Immune*					
CRS	8 (66.7)	0 (0)	0	0	8 (66.7)
ICANS	0	0	0	0	0
*Hematologic*					
Leukopenia	0	4 (33.3)	4 (33.3)	4 (33.3)	12 (100)
Neutropenia	2 (16.7)	1 (8.3)	6 (50)	2 (16.7)	11 (91.7)
Anemia	1 (8.3)	6 (50)	4 (33.3)	0	11 (91.7)
Thrombocytopenia	0	5 (41.7)	1 (8.3)	0	6 (50)
Lymphopenia	0	0	2 (16.7)	10 (83.3)	12 (100)
Coagulopathy	0	0	1 (8.3)	0	1 (8.3)
Blood fibrinogen decreased	1 (8.3)	0	1 (8.3)	0	2 (16.7)
*Gastrointestinal*					
Nausea	1 (8.3)	1 (8.3)	0	0	2 (16.7)
Vomiting	0	2 (16.7)	1 (8.3)	0	3 (25)
Stomach pain	0	2 (16.7)	0	0	2 (16.7)
Gastric hemorrhage	0	2 (16.7)	0	0	2 (16.7)
*Infection*					
Pneumonia	0	1 (8.3)	1 (8.3)	0	2 (16.7)
*Metabolism and nutrition*					
Hypokalemia	3 (25)	1 (8.3)	0	1 (8.3)	5 (41.7)
Decreased appetite	0	4 (33.3)	0	0	4 (33.3)
Electrolyte imbalance	0	2 (16.7)	0	0	2 (16.7)
Hypertriglyceridemia	0	1 (8.3)	1 (8.3)	0	2 (16.7)
Hypoalbuminemia	0 (0)	7 (58.3)	0	0	7 (58.3)
Hypocalcemia	2 (16.7)	1 (8.3)	0	0	3 (25)
Hypophosphatemia	1 (8.3)	1 (8.3)	0	0	2 (16.7)
*Hepatic*					
Gamma-glutamyl transferase increased	0	1 (8.3)	0	0	1 (8.3)
Alkaline phosphatase increased	0	0	1 (8.3)	0	1 (8.3)
Aspartate aminotransferase increased	1 (8.3)	0	1 (8.3)	0	2 (16.7)
Blood bilirubin increased	0	1 (8.3)	0	0	1 (8.3)
*Skin and subcutaneous tissue*					
Skin ulcer	0	1 (8.3)	0	0	1 (8.3)
Pruritus	1 (8.3)	0	0	0	1 (8.3)
*Renal and urinary*					
Hematuria	1 (8.3)	0	0	0	1 (8.3)
*Other*					
Weight decreased	1 (8.3)	2 (16.7)	0	0	3 (25)
Insomnia	0	2 (16.7)	0	0	2 (16.7)
Cough	0	1 (8.3)	0	0	1 (8.3)
Loin pain	0	1 (8.3)	0	0	1 (8.3)
Malaise	1 (8.3)	0	0	0	1 (8.3)

*CRS* cytokine release syndrome, *ICANS* immune effector cell-associated neurotoxicity syndrome

Efficacy was assessed on day 28 postinfusion in 10 patients (excluding Patients 5 and 12). Three GC/GEJC patients achieved a partial response (PR) at the initial evaluation, although Patient 8 later progressed to progressive disease (PD) at the time of confirmation. Seven patients exhibited tumor shrinkage (Fig. 3b). The overall response rate (ORR) and disease control rate (DCR) for the cohort were 20.0% (95% CI 2.5–55.6) and 80.0% (95% CI 44.4–97.5), respectively (Table 2). Among the 8 GC/GEJC patients, the ORR and DCR were 25.0% (95% CI 3.2–65.1) and 75.0% (95% CI 34.9–96.8), respectively. For GC/GEJC patients with medium-to-high levels of CLDN18.2 expression (*n* = 6; criteria in Supplementary Table 1^a^), the ORR and DCR were 33.3% (95% CI 4.3–77.7) and 100% (95% CI 54.1–100), respectively, and the median progression-free survival (PFS) and overall survival (OS) were 148 (95% CI 60.6–235.4) and 327 (95% CI 131.4–522.6) days, respectively. The best overall responses included 2 PR patients and 6 stable disease (SD) patients (Supplementary Table 2). Circulating tumor cell (CTC) levels were found to decrease postinfusion in select patients (Fig. 3d). Notably, the lesions of Patient 8 demonstrated marked reduction or near-complete resolution and were unmeasurable at 4 weeks postinfusion according to computed tomography (CT) (Fig. 3e).

**Table 2:** Responses of different tumor types according to RECIST version 1.1

Tumor type	No. of patients	CR (%)	PR (%)	SD (%)	PD (%)	ORR, *n* (%) [95% CI]	DCR, *n* (%) [95% CI]	mPFS, (day) [95% CI]	mOS, (day) [95% CI]
GC/GEJC	8	0	2 (25)	4 (50)	2 (25)	2 (25) [3.2–65.1]	6 (75) [34.9–96.8]	85 [0.0–208.3]	232 [14.4–449.6]
PC	2	0	0	2 (100)	0	0	2 (100) [15.8–100]	NA	174 NA
GC/GEJC with medium-high Claudin18.2 expression	6	0	2 (33.3)	4 (66.7)	0	2 (33.3) [4.3–77.7]	6 (100) [54.1–100]	148 [60.6–235.4]	327 [131.4–522.6]
All	10	0	2 (30)	6 (60)	2 (20)	2 (20) [2.5–55.6]	8 (80) [44.4–97.5]	148 [0.0–300.5]	232 [106.5–357.5]

*CR* complete response, *PR* partial response, *SD* stable disease, *PD* progressive disease, *ORR* overall response rate, *DCR* disease control rate, *mPFS* median progression-free survival, *mOS* median overall survival, *NA* not applicable

CAR-T cell dynamics were monitored via qPCR. Each patients displayed CAR-T cell proliferation after infusion, and the median duration of CAR-T cell persistence in peripheral blood was 10 days (range: 10–54 days) after the initial infusion (Supplementary Fig. 3c). At 54 days postinfusion, no expansion of CAR-T cells was observed in any of the patients. The median peak CAR copy number was 12,906 copies/μgDNA (range: 2657.9–47,704.77). Compared with PD patients, PR patients presented significantly greater peak CAR copy numbers (Fig. 3f). Elevated IL-7 and XCL1 levels were also more common among patients with a PR than among those with PD/SD (Fig. 3g).

### Analysis of RD07 dynamics and therapeutic efficacy

**Fig. 4:**
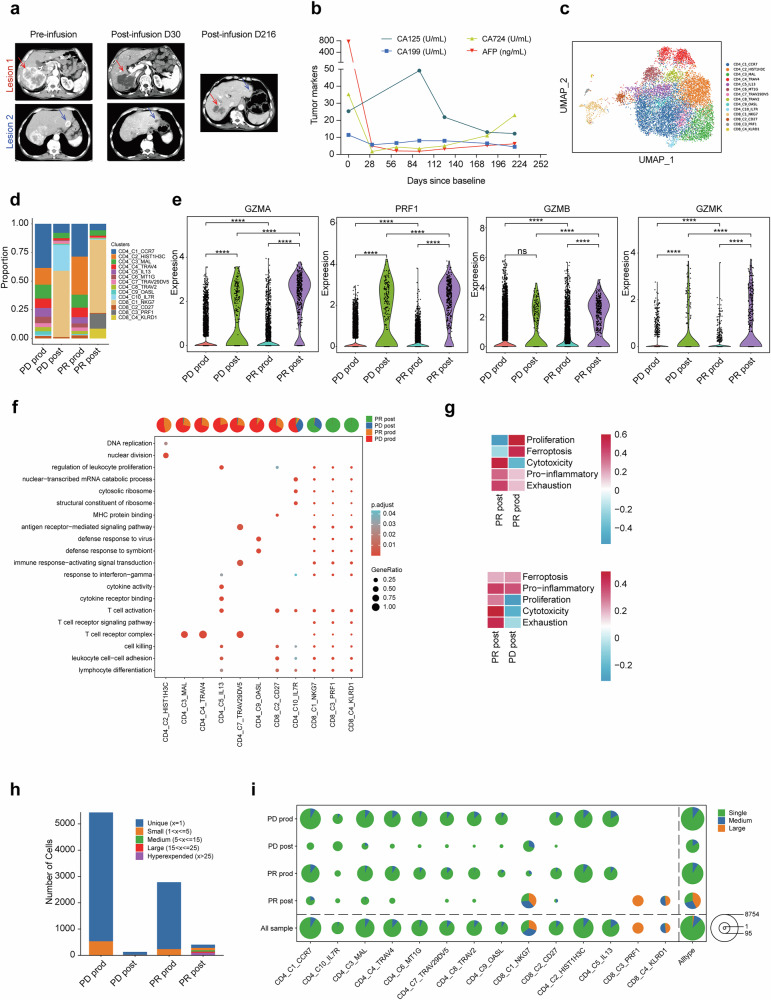
Comparative analysis of RD07 cell dynamics and therapeutic efficacy in PR versus PD patients pre- and postinfusion. **a** CT scans reveal a reduction in the tumor lesion of Patient 2 (PR patient) following RD07 infusion. **b** The expression levels of tumor markers in Patient 2 decreased after treatment. **c** UMAP feature representation of 11,853 CAR-T cells combined from two patients, identifying 14 distinct clusters of T cells on the basis of conventional markers. Color-coded by clusters and cell subsets as indicated. **d** Bar plots illustrating the relative abundance of each T-cell cluster in the products and engrafted CAR-T cells of patients with a PR or PD. Prod, product. **e** Expression levels of cytotoxicity-related genes in CAR-T cell products and engrafted CAR-T cells from patients with PR or PD. **f** Key pathways associated with each T-cell cluster from PR and PD patients revealed by GO enrichment analysis. **g** GSVA enrichment analysis highlighting differences in functional pathways between PR patients and PD patients. **h** The number of cells associated with different TCR clonotypes. **i** Pie chart displaying the percentages of different TCR clonotypes across different T-cell clusters. Statistical analysis was performed via unpaired two-tailed Wilcoxon–Mann–Whitney *U* tests. ****P < 0.0001

Patient 2, a 57-year-old man with GC, received five RD07 infusions over 6 months after failure to respond to more than 26 months of prior therapies (neoadjuvant chemotherapy, surgery, adjuvant chemotherapy, anti-PD-1, and tyrosine kinase inhibitors). Transient CAR-T cell expansion occurred after the first two infusions, which correlated with sustained regression of liver metastases and decreased tumor marker levels until day 216. The patient ceased further imaging after this timepoint and died on day 466 (Fig. 4a, b).

To investigate the mechanisms underlying RD07 efficacy, we conducted scRNA-seq on CAR-T cell products as well as CAR-T cells isolated from the postinfusion peripheral blood of patients. 2 (PR) and 3 (PD). Uniform manifold approximation and projection (UMAP) was performed for the 11,853 cells, which identified ten CD4^+^ and four CD8^+^ clusters (Fig. 4c), with distinct cellular proportions between PR patients and PD patients (Fig. 4d). Compared with the pretreatment product and PD patient samples, the posttreatment PR samples presented increased expression of cytotoxicity-related genes (*PRF1* and *GZMA/B/K*) (Fig. 4e). Pathway enrichment analysis demonstrated that the CD8-*PRF1/KLRD1* clusters were significantly enriched in signaling pathways associated with T-cell activation and killing pathways (Fig. 4f). Moreover, GSVA revealed increased cytotoxicity, proliferation, and exhaustion pathways in the PR group relative to the PD group (Fig. 4g). TCR clonal expansion (small/large/hyperexpanded clones; Fig. 4h) and amplification of CD8-*NKG7/PRF1* clusters (Fig. 4i) further underscored the superior antitumor activity in the PR patient.

### RD07 enhances endogenous T-cell clonal diversity and activation in the TME

**Fig. 5:**
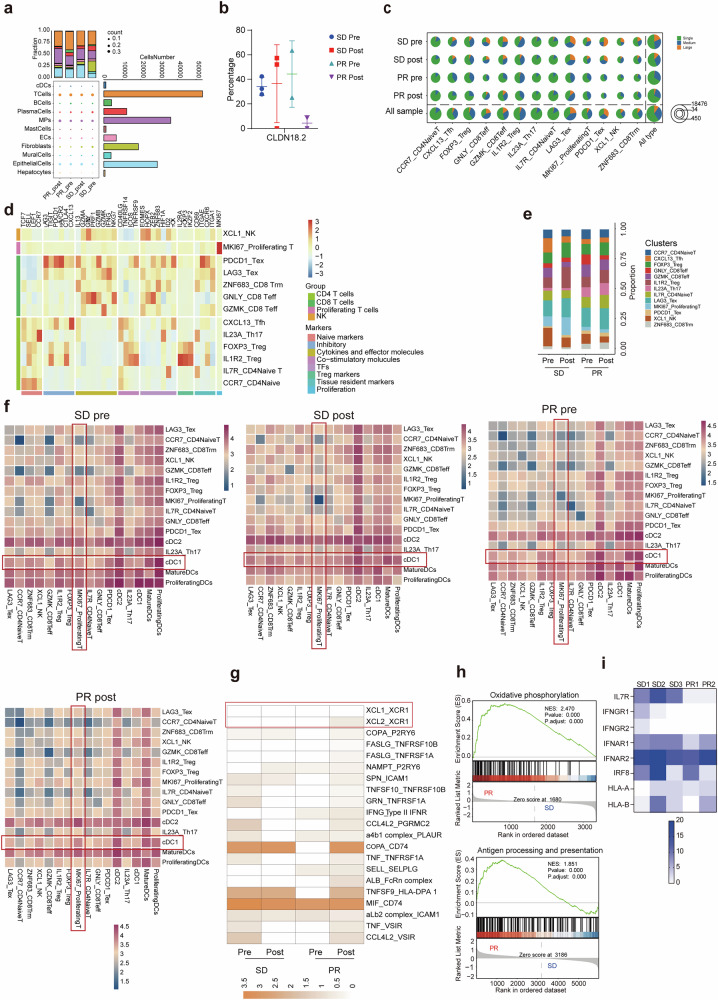
scRNA-seq analysis of tumor tissues from patients with gastric cancer treated with RD07. **a** Cell lineage compositions of tumor samples from patients with SD and PR before and after RD07 treatment. The size of each circle represents the proportion of specific cell lineages/types. **b** Percentage of CLDN18.2 in patient samples before and after RD07 therapy. **c** Pie chart illustrating the percentage of TCR clonotypes across different cell subtypes. **d** Heatmap displaying the average expression of selected genes associated with T-cell function in each cell cluster. **e** Bar plot of 13 immune cell clusters in patients before and after treatment. **f** Heatmap illustrating the logarithmic changes in interacting receptor‒ligand pairs between different cell types in patients before and after RD07 therapy. The darker the color is, the stronger the interaction between the interaction pairs. **g** Heatmap illustrating important interaction pairs between Ki67^+^ proliferating T cells and cDC1s in PR patients. **h** GSEA revealed pathways related to oxidative phosphorylation in Ki67^+^ T cells and antigen processing and presentation in cDC1s in PR patients. **i** Heatmap showing SNP mutation counts in *IL-7R*-, *HLA-* and *IFN*-related genes in SD and PR patients. Statistical analysis was performed via unpaired two-tailed Wilcoxon–Mann–Whitney *U* tests

To evaluate RD07’s therapeutic impact across clinical outcomes, scRNA-seq was conducted on paired GC biopsy specimens derived from five patients (3 SDs and 2 PRs, pre- and posttreatment; Supplementary Table 3). After quality filtering, 246,107 cells were analyzed, and 47 unique cell types were identified via UMAP (Supplementary Fig. 4a). These cells clustered into eleven major lineages dominated by T cells (consistent with prior studies,^27^ followed by mononuclear phagocytes (MPs) and epithelial cells. Posttreatment, patients with SD presented reduced proportions of B cells, plasma cells, and fibroblasts alongside increased proportions of mast cells, epithelial cells, and endothelial cells (ECs). In contrast, PR patients showed only a B-cell decline, highlighting interpatient heterogeneity (Fig. 5a). Notably, the proportion of CLDN18.2^+^ tumor cells decreased significantly in PR patients (Fig. 5b), confirming RD07’s targeting specificity.

Preclinical evidence suggests that IL-7/XCL1 enhances CAR-T cell activity by recruiting cDC1s and activating endogenous T cells. While the abundance of cDC1s did not differ between the PR and SD groups (likely due to their low baseline frequency), their functional impact may be amplified through endogenous T-cell activity. TCR clonal analysis revealed a remarkable increase in the clonal diversity and expansion of *GNLY/GZMK*^+^ CD8^+^ effector T (Teff) and *LAG3*^+^ exhausted T (Tex) cells in PR patients posttreatment (Fig. 5c), which aligns with the systemic immune activation mechanism of ExCAR-T cells.

### TME cellular crosstalk enhancement by RD07

Reanalysis of GC tumor-infiltrating immune cells revealed thirteen T/NK subpopulations, including five CD8^+^ T-cell clusters, six CD4^+^ T-cell clusters, and proliferating T/NK subsets (Fig. 5d, e). Post-RD07, PR patients presented enrichment of *GZMK*^+^CD8^+^ Teff, *IL23α*^+^Th17, *IL-7R*^+^CD4^+^ naïve T, and *ZNF683*^+^CD8^+^ tissue-resident memory T (Trm) cells, all of which were more abundant in PR patients than in SD patients before treatment (Fig. 5e). These clusters presented upregulated expression of naïve markers, cytokines, effector molecules, costimulatory receptors, and proliferation/tissue retention markers (Supplementary Fig. 4b).

Gene set variation analysis (GSVA) revealed increased T-cell cytolytic activity, MP endocytosis, and cDC1 type I interferon signaling in PR patients (Supplementary Fig. 5a–g), indicating an inflammatory TME. CellPhoneDB analysis revealed enhanced post-treatment communications between MPs and fibroblasts in PR group, and elevated interactions between stromal cells and immune subsets in SD group (Supplementary Fig. 5h). Mirroring murine findings, PR patients presented increased *Ki67*^+^ proliferating T-cell-cDC1 interactions (Fig. 5f), driven by upregulated *XCL2-XCR1* signaling, TNF superfamily ligands, and IFN-γ pathways (Fig. 5g). PR patients also exhibited enriched oxidative phosphorylation in *Ki67*^+^ T cells and enhanced antigen presentation in cDC1s (Fig. 5h). Whole-exome sequencing (WES) revealed higher *IL-7R* mutation rates in SD patients than in PR patients, although the rates of HLA and IFN pathway mutations were comparable (Fig. 5i; Supplementary Table 4). Taken together, these findings indicate that RD07 remodels the TME by promoting T-cell–cDC1 crosstalk via IL-7/XCL1 coexpression.

### Spatial reconfiguration of the TME post-RD07

**Fig. 6:**
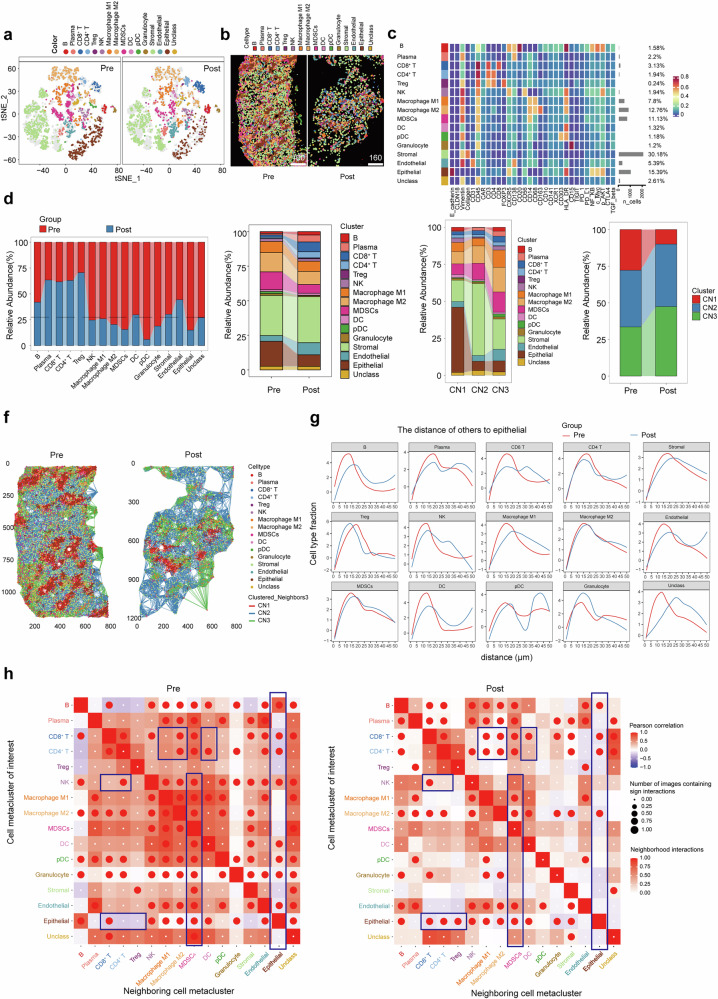
Imaging mass cytometry analysis of the spatial landscapes of human gastric cancer tissues before and after RD07 treatment. **a** t-SNE visualization of cell types, illustrating the distribution of single cells in slices. **b** Cell type visualized with pseudocolor in pre- and posttreatment sample slices. **c** Heatmaps were generated to visualize the expression profiles of specific markers across individual cell types, and the proportional distribution of each cell type population. **d** Abundances of different cell types in pre- and posttreatment samples. **e** Proportions of cell types within three clusters (tumor (CN1), stromal (CN2), and immune (CN3)) and their neighboring clusters across the two samples. **f** Mapping of the immune microenvironment network, including a color code for 16 cell types and the colored line indicating three clusters. **g** To assess the spatial association of various cell types with epithelial cells, we calculated a proximity-based fraction for each cell type. This metric represents the number of cells of that type found within a 1 µm radius of epithelial cells, divided by the overall number of that cell type present in the entire slice. **h** Pearson correlation analysis of the cell interactions before and after treatment

Imaging mass cytometry (IMC) of Patient 9’s tumor (from the same site pre/posttreatment) with a 32-plex antibody panel was used to analyze the spatial landscape changes in cancer tissues, contingent upon the eligibility of the tumor sample. The tumor shrunk by 21.86% after RD07 treatment, but the tumor cells almost disappeared from pathology, and the sample cells appeared sparse (Supplementary Fig. 7a–d). A total of 7100 cells were classified into 31 different clusters (details in Supplementary Table 5) via a supervised lineage assignment method (Supplementary Fig. 7c, d), which resulted in the identification of 16 cell types. Post-RD07, the proportion of T/B lymphocytes increased, whereas the proportions of M2 macrophages, MDSCs, and tumor cells decreased (Fig. 6a–d), which aligns with the murine and clinical data. Downregulation of *c-Myc/CXCR5* in MDSCs/tumor cells suggested reduced migratory potential.^28^ Decreased *PD-1/PD-L1* expression and modulation of the *NF-κB/TGF-β* pathways indicated TME immunostimulation (Supplementary Fig. 7e).^29^

Neighborhood analysis partitioned the TME into tumor (CN1), stromal (CN2), and immune (CN3) niches. Posttreatment, CN1 was reduced, with strengthened T-cell–NK cell/M1 macrophage/DC interactions and diminished MDSC crosstalk (Fig. 6e–h). These spatial shifts reflect tumor necrosis and TME remodeling, reinforcing RD07’s immunoregulatory potential in digestive cancers.

## Discussion

The efficacy of CAR-T cell therapy against solid tumors is constrained by the intrinsic biological characteristics of the tumors themselves (tumor heterogeneity and complex TME) and CAR-T cell features (low survival and expansion capacities).^9,30^ In this study, we introduced IL-7 and XCL1 into a CAR construct to overcome these obstacles in digestive cancers. Although T cells express a basal level of XCL1, the level is insufficient to exert a potent tumor control effect (Fig. 1f, n). A recent investigation has shown that CAR-T cells genetically engineered for the concurrent expression of IL-7 and CCL19 undergo safe expansion and long-term persistence in patients with relapsed or refractory large B-cell lymphoma, a finding validated in both preclinical models and clinical trials.^31^ In our study, tumor suppression by ExCAR-T cells was demonstrated in GC and PC mouse models. Mechanistically, ExCAR-T cell therapy increased the number of DCs and endogenous T cells, resulting in a “hot” TME. These finding was in line with the outcomes of a prior investigation.^32^ Moreover, we confirmed the efficacy and safety of RD07 in clinical trials and demonstrated that the expression of medium-to-high CLDN18.2 in patients with DTC was associated with a good clinical response. ScRNA-seq and IMC analysis further confirmed that IL-7 and XCL1 enhanced CAR-T cell efficacy through systemic immune activation and remodeling of the TME.

In our immunocompetent mouse studies, CAR signaling domains were constructed primarily via murine components, with the exception of the human-origin 4-1BB endodomain, which was chosen to optimize the function and persistence of CAR-T cell while minimizing immunogenic responses.^33^ In the CLDN18.2 model, both cCAR-T cells and ExCAR-T cells remained detectable day 21 after treatment, with more than 140-fold more ExCAR-T cells than cCAR-T cells. By 49 days posttreatment, the abundance of ExCAR-T cells in the tumor was elevated by more than 600 folds, indicating that the use of a human 4-1BB endodomain does not induce significant immunogenicity. In the Pan02^CD19^ tumor model, the elimination of B cells prevents the production of antibodies against the murine tumor line, indicating that the observed protection is not the result of a vaccination-like response. In our rechallenge model, we used a tumor lacking the CAR-specific antigen, which means that CAR-T cells themselves would not directly control tumor growth in this context. These findings strongly suggest that the long-term immunity observed is due to the involvement and activation of endogenous T cells rather than the direct action of CAR-T cells.

While XCL1 has been implicated in Treg generation, its impact is highly context dependent and varies significantly with specific diseases and the local immune environment.^34^ In our ExCAR-T design, XCL1 exerts a coordinated effect with IL-7 in a synergistic manner, thereby shaping a TME conducive to the functional exertion of cytotoxic T cells. The combination is intended to amplify the immune-stimulatory capacity of XCL1, thereby optimizing its role in promoting robust antitumor activity. We observed a marked increase in CD8^+^ and CD4^+^ T cell infiltration in posttreatment tumor samples in our study (Fig. 6a, d). This is accompanied by a modest increase in Treg cells, while TGF-β expression in Treg cells tends to decrease in posttreatment tumor samples (Supplementary Fig. 7e). These findings suggest favorable modulation of the immune environment.

Prior investigations have elucidated the cytotoxic mechanisms underlying the anti-tumor activity of CAR-T cells against their target cells.^35^ They demonstrated the CAR-T cells at the peak period expressed genes associated with proliferation and cytotoxicity. Additionally, CAR-T cells are engineered for the expression of cytokines and chemokines to improve antitumor activity by increasing host immunity.^36^ Our study expands these discoveries to DTCs, and we identified a mechanism by which endogenous T cells and CAR-T cells are activated in vivo through the upregulation of cytotoxicity-related genes and pathways in the PR group after RD07 treatment. Moreover, the interaction of cDC1s with CD8^+^ T cells is positively correlated with the efficacy of cancer immunotherapy.^37^ In our study, we demonstrated that the interaction between DCs and T cells was enhanced after RD07 treatment, which was associated with a better clinical response. The primary limitation of CAR-T cell therapy in solid tumors is the immunosuppressive TME, such as myeloid cells, and the secretion level of TGF-β.^38^ Using IMC analyses, we demonstrated that immunosuppressive signals, including TGF-β expression and the proportions of MDSCs and M2 macrophages, were decreased in the TME, indicating that RD07 reshaped the TME via IL-7 and XCL1 coexpression. The IL-7/IL-7R signaling cascade exerts a pivotal regulatory role in the differentiation and functional modulation of lymphocytes.^39^ Most deleterious mutations in IL7RA are associated with immunodeficiency and inflammatory disease.^40^ An interesting finding of our work is the identification of SNPs in *IL-7R* in patients before treatment and the finding that patients with SD presented a greater frequency of missense mutations. However, the correlation between IL7R gene mutations and the therapeutic efficacy of CAR-T cell therapy warrants further in-depth investigation.

A prior investigation demonstrated a positive correlation between the clonal proliferation of T cells and favorable clinical outcomes in patients following CAR-T cell therapy.^41^ Consistent with this, we found that TCR clone diversity increased and that clonal amplification of the CD8^+^ Teff and *LAG3*^+^ Tex subtypes occurred in the TME of patients; these effects were observed for the CD8 PRF1 and KLRD1 clusters of CAR-T cells in one PR patient. These results are consistent with the transcriptomic data demonstrating that endogenous T cells and CAR-T cells exhibit enhanced antitumor activity in patients with a positive treatment response. Clones exhibit different patterns of T-cell expansion in vivo, suggesting that these cells have a strong capacity to proliferate and survive.^42^ Therefore, different CAR-T cell clones were generated in patients who achieved a PR after treatment, which may have promoted a better outcome for these patients.

A recent study of CLDN18.2-targeted CAR-T cells for patients of chemotherapy-refractory gastrointestinal cancers^17^ reported an ORR of 38.8%, a DCR of 91.8%, a median PFS of 4.4 months, and a median OS of 8.8 months. This cohort comprised patients with favorable characteristics: ECOG performance status 0–1 (100%), CLDN18.2 medium/high expression (91.8%), ≥3 prior lines of therapy (23%), and ≥3 metastatic organs (41%). In contrast, our trial enrolled a more advanced population with the following features: ECOG score of 0–1 (50%) and 2 (50%), medium/high CLDN18.2 expression (66.6%), ≥3 prior lines (50%), and ≥3 metastatic organs (50%), with no bridging therapy administered. The outcomes were an ORR of 20%, a DCR of 80%, an mPFS of 148 days, and an mOS of 232 days. While these results appear less favorable than those of the reference trial, we attribute this disparity to our inclusion of more terminally ill patients with aggressive disease. Notably, 7/10 patients exhibited tumor regression. Subgroup analysis of patients with medium/high CLDN18.2 expression (*n* = 6) revealed improved outcomes: ORR, 33.3%; DCR, 100%; mPFS, 148 days; and mOS, 327 days. This subgroup derived the greatest benefit from RD07, which was encouraging for refractory advanced digestive cancers in clinical therapeutics. Our findings align with current CLDN18.2-targeted trial criteria^43^ and suggest that restricting enrollment to patients with medium/high expression level of CLDN18.2 may optimize therapeutic efficacy. The small cohort size (*n* = 10) limits statistical power, necessitating validation in larger trials. A limitation of this study lies in the lack of head-to-head comparisons regarding the therapeutic efficacy between second-generation CAR-T cells and ExCAR-T cells within clinical trial settings. Evaluating the effectiveness of ExCAR-T cells in patients who are refractory to second-generation CAR-T cell treatment would offer more compelling insights and strengthen the conclusions of our research.

In conclusion, the appropriate combination of immunoregulatory factors, including IL-7 and XCL1, could enhance the antitumor response to CAR-T cell therapy and the interplay between T cells and DCs to remodel the tumor microenvironment. Our data indicate that treatment with RD07 cells yielded a promising clinical response with acceptable safety in digestive tract cancer patients with medium to high expression level of CLDN18.2. Furthermore, validation and investigation in large, randomized controlled studies are essential.

## Materials and methods

### Mice and cell lines

Both female and male B-NDG mice (NOD.CB17-Prkdcscid Il2rg^tm1^) were supplied by a Beijing commercial supplier in China (Biocytogen). C57BL/6 and BALB/c mice, aged 7–10 weeks, were obtained from a laboratory animal supplier in Hangzhou, China (Ziyuan). Animals were maintained under pathogen-controlled conditions at Raygen Health Molecular Medicine Technology Co., Ltd. All animal-related studies were reviewed and authorized by the institutional ethics committee of the PKU–Nanjing Joint Institute of Translational Medicine (IACYC-2022-014).

The pancreatic adenocarcinoma cell line Pan02 with a C57BL/6 genetic background and expresses human Claudin18.2 or murine CD19, was constructed by Nanjing Bioheng Biotech Co., Ltd. The colon carcinoma cell line CT26 (NCACC), which has a BALB/c genetic background and expresses human Claudin18.2 or human EGFRvIII. For in vitro studies, MutuDC 1940 cells and Pan02 cells were expanded in RPMI-1640 medium supplemented with fetal calf serum (10%), penicillin (100 U/mL), streptomycin (100 µg/mL), L-glutamine (2 mM), 2-mercaptoethanol (50 µM), and HEPES (25 mM). In contrast, CT26 cells were propagated in DMEM containing 10% fetal calf serum and standard antibiotic supplementation.

### Preparation of human CAR-T cells

The Claudin18.2 VHH was combined with the CD8α transmembrane domain and 4-1BB/CD3ζ intracellular regions to construct a second-generation CAR, which was inserted into the MSCV vector. To enable co-expression of human IL-7 and XCL1 along with the CAR, the coding sequences were separated by a 2A peptide. All sequences were synthesized at General Biol. CAR expression in human T cells was achieved by retroviral transduction. Retroviral supernatants encoding the CAR construct were prepared according to the procedures described above and applied for human T-cell transduction. Human PBMCs were activated for 48 h using CD3/CD28 Dynabeads (Thermo Fisher Scientific) in the presence of recombinant IL-2 (Jiangsu Sihuan Bioengineering Co., Ltd.), followed by CAR gene delivery as described above. The cells were subsequently cultured for an additional 7 days with IL-2 (200 IU) for subsequent experiments.

### In vivo models for assessing the anti-tumor effects of human CAR-T cells

B-NDG mice were subcutaneously implanted with 4 × 10⁶ NUGC4 tumor cells expressing human CLDN18.2 on the right flank on day 0. On day 25, 5 × 10⁵ human CLDN18.2 CAR-T cells were administered intravenously. Tumor progression and mouse survival were monitored, and in selected experiments, peripheral blood was collected to quantify CAR-T cells (123 count eBeads, Thermo Fisher).

### Clinical trial design

This was a single-arm, open-label, dose-escalation study to evaluate the safety and efficacy of RD07 in patients with advanced gastric and pancreatic tumors (www.chictr.org.cn, ChiCTR2000038650). The enrollment criteria included the following: (1) aged 18–69 years inclusive (sex not limited); (2) advanced GC/GEJC and PC confirmed by pathology as CLDN18.2-positive; (3) ineffective second-line treatment or failure of first-line treatment and unwillingness to receive other treatment; (4) one or more measurable lesions as defined by RECIST 1.1; (5) ECOG score 0–2; (6) absolute neutrophil count (ANC) ≥ 1.5 × 10^9^/L, absolute platelet count (APC) > 60 × 10^9^/L; (7) serum total bilirubin < 51 µmol/L, serum alanine aminotransferase (ALT) and aspartate transaminase (AST) < 3 times the upper limit of normal (ULN), serum creatinine < 176.8 µmol/L; (8) echocardiographic examination revealed a left ventricular ejection fraction (LVEF) of more than 50%; (9) absent of active pulmonary infection; (10) life expectancy of more than 3 months; (11) ability to tolerate apheresis; and (12) capacity to give informed consent. The exclusion criteria were as follows: (1) allergy to any of the components of the cell products; (2) previously received antibody drugs or cell therapy targeting Claudin18.2; (3) uncontrolled intracranial metastatic lesions; (4) failed to achieve recovery from prior antitumor treatment or related toxicities, with the exception of alopecia; (5) active upper gastrointestinal disease within 4 weeks, such as bleeding or ulcers; (6) received antitumor therapy within 2 weeks of preapheresis, including chemotherapy, radiotherapy, targeting and immunotherapy; (7) current systemic corticosteroid therapy; (8) history of severe heart disease; (9) history of severe cerebrovascular accidents; (10) ECG indicating a prolonged QT interval or severe arrhythmia; (11) poorly controlled hypertension or diabetes; (12) uncontrolled active infection (excluding uncomplicated urinary tract infection and bacterial pharyngitis); and (13) active infection with HCV, HIV, syphilis and HBs-Ag positive or HBV DNA exceeding the ULN; (14) history of other primary cancers; (15) autoimmune diseases requiring treatment or requiring immunosuppressive therapy; (16) history of alcoholism, drug abuse or mental illness; and (17) participation in another clinical trial within 2 weeks before screening; (18) for women, being pregnant, lactating, or fertile and unable to take effective contraceptive measures; (19) moderate or greater amount of ascites, or progressively increasing ascites; (20) any other trait that might increase subject risk and affect the study results. This clinical trial obtained ethical approval from the Research Ethics Committee for Clinical Studies of the First Affiliated Hospital of Zhengzhou University (L2020-Y158-001). Before enrollment in the clinical trial, all patients provided written informed consent in line with the ethical principles of the Declaration of Helsinki. From January 2021 to February 2023, 12 patients from the First Affiliated Hospital of Zhengzhou University were enrolled in this trial, and their baseline characteristics are shown in Supplementary Table 1. The effectiveness of these novel CAR-T cells was evaluated in ten patients. The clinical study process is shown in Supplementary Fig. 3a.

### CAR-T cell treatment and evaluation

No bridging therapy was administered before the CAR-T cell infusion. Lymphodepleting chemotherapy regimen was composed of cyclophosphamide, fludarabine with or without nab-paclitaxel before cell infusion for 3‒5 days. The use of these agents depended on each patient’s condition, and all relevant results are included in Supplementary Table 2. Patients subsequently received 0.1–2 × 10^7^ CAR-T cells/kg weight, and an accurate dose is shown in Supplementary Table 2. This clinical trial was primarily assess the safety profile of RD07 for the treatment of advanced CLDN18.2-positive GC, GEJC and PC. Treatment toxicity was evaluated by physical and clinical laboratory examinations and assessed by the Common Terminology Criteria for Adverse Events (CTCAE) version 5.0. CRS and ICANS were graded according to the American Society for Transplantation and Cellular Therapy (ASTCT) criteria. Tumor responses to CAR-T cell treatment were evaluation based on RECIST 1.1 criteria both pre- and post-infusion.

### Single-cell RNA-seq

Tumor samples were collected from five GC patients before and after RD07 treatment (Supplementary Table 3). Single-cell suspensions were prepared and subjected to scRNA-seq library construction using the GEXSCOPE single-cell RNA library kit (Singleron Biotechnologies) following the protocol. Pools were sequenced on an Illumina HiSeq X platform with 150 bp paired-end reads. Raw reads were quality-filtered using FastQC and fastp, followed by removal of poly-A tails and adaptor sequences using Cutadapt. Clean reads were then aligned to the GRCh38 reference genome (Ensembl v92 annotation) with STAR, and gene/UMI counts were quantified using featureCounts to generate expression matrices for downstream analyses. CAR-T cell products and CAR-T cells at day 10 after infusion were collected via a MoFlo-XDP cell sorter (Beckman Coulter, Brea, CA) by staining with CD3 and MonoRab Tm rabbit anti-camelid VHH cocktail. Single-cell TCR-seq and 5′ gene expression profiling were subsequently performed on the same suspension using the Chromium Single Cell V(D)J Solution (10× Genomics) following the manufacturer’s instructions.^44^

### TCR analysis

TCR clonotype annotation was implemented using the Cell Ranger (v4.0.0) VDJ pipeline, with the GRCh38 reference genome as the analytical template. Briefly, a TCR diversity index integrating clonotype frequency and barcode details was constructed. For subsequent TCR profiling, only cells harboring a single functional TCR α-chain (TRA) and a single functional TCR β-chain (TRB) were filtered for downstream analysis. Each distinct TRA/TRB pairing was designated as an individual clonotype. Clonal cells were defined as those cell populations in which the identical clonotype was identified in no fewer than two cells, and the clonality extent was quantified by the cell count sharing an identical clonotype.

### IMC staining and analysis

Formalin-fixed, paraffin-embedded tumor tissues from Patient 9 collected before and 30 days after RD07 infusion were obtained from the Pathology Department of hospital. Sections were deparaffinized, rehydrated through a graded ethanol series, and subjected to antigen retrieval, followed by blocking with 3% BSA. A pre-validated antibody cocktail (Supplementary Fig. 6b) was applied and incubated overnight at 4 °C. After washing, nuclei were labeled with Intercalator-Ir, and slides were air-dried prior to acquisition on the Hyperion™ Imaging System. IMC datasets were visualized using MCD Viewer, exported as 16-bit .ome.tiff images, and processed in CellProfiler to generate single-cell segmentation masks. Single-cell marker intensities were extracted in HistoCAT, and batch effects were corrected using Harmony. We used Harmony v0.1.0 to eliminate batch effects. Rphenograph v0.99.1 was used for clustering with “*K* = 45” and it is also available on github (https://github.com/JinmiaoChenLab/Rphenograph). Clustering results were summarized using median *z*-score heatmaps, and phenotypic heterogeneity was examined by t-SNE. Spatial distribution and neighborhood interactions were further evaluated using Cytomapper and imcRtools.

### Statistical analysis

Statistical tests were performed via GraphPad Prism version 9. The results are expressed as the mean ± SEM. The unpaired two-tailed Wilcoxon–Mann–Whitney *U* test or two-sided unpaired *t* tests was employed to assess the statistical significance of differences between two independent sample groups. For experiments with more than two groups, statistical analyses were performed via one- or two-way ANOVA. For preclinical studies, survival was measured via the Kaplan‒Meier method. For the analyses of PFS and OS, CAR-T cells infusion was the starting point. Across all figures, statistical significance is indicated as follows: *P < 0.05, ** *P* < 0.01, *** *P* < 0.001, **** *P* < 0.0001 with the threshold for statistical significance defined at *P* < 0.05.

## Supplementary information

Supplementary Materials

## Data Availability

The raw data of scRNA-seq and TCR-seq for this study can be found in the GSA database with the BioProject number: PRJCA046448 (submission is: HRA016029, please access it from the following link: https://ngdc.cncb.ac.cn/gsa-human/browse/HRA016029). All other datasets generated during the course of this study are available from the corresponding author upon reasonable request.
