# Supplementary material for: Efficacy and immunomodulatory effect of Claudin18.2-specific IL-7/XCL1 armored CAR-T cells in digestive tract cancer: preclinical and clinical analysis
Source: Signal Transduct Target Ther. 2026 Mar 9;11:87. doi: 10.1038/s41392-026-02621-8 (PMC12972057; doi:10.1038/s41392-026-02621-8)
Supplement: Supplementary file 1 — Supplementary Materials [file 41392_2026_2621_MOESM1_ESM.docx]

Supplementary Materials for

**Efficacy and immunomodulatory effect of Claudin18.2-specific IL-7/XCL1 armored** **CAR-T cells in digestive tract cancer: preclinical and clinical analysis**

Xuan Zhao^1†^, Jinyan Liu^1†^, Zhen Zhang^1†^, Yali Zhou^2†^, Shuiling Jin^3^, Hong Zong^3^, Feng Wang^3^, Min Song^3^, Yali Zhong^3^, Qinglong Li^4^, Bo Pei^2^, Yong Yu^2^, Ming Gao^2^, Wengang Ge^2^, Lu Han^2^, Jiangtao Ren^2*^ and Yi Zhang^1,5,6,7*^

Correspondence to: [yizhang@zzu.edu.cn](mailto:yizhang@zzu.edu.cn) and jiangtao.ren@bioheng.com

**This PDF file includes:**

Figures. S1 to S7

Tables S1 to S5

Materials and methods


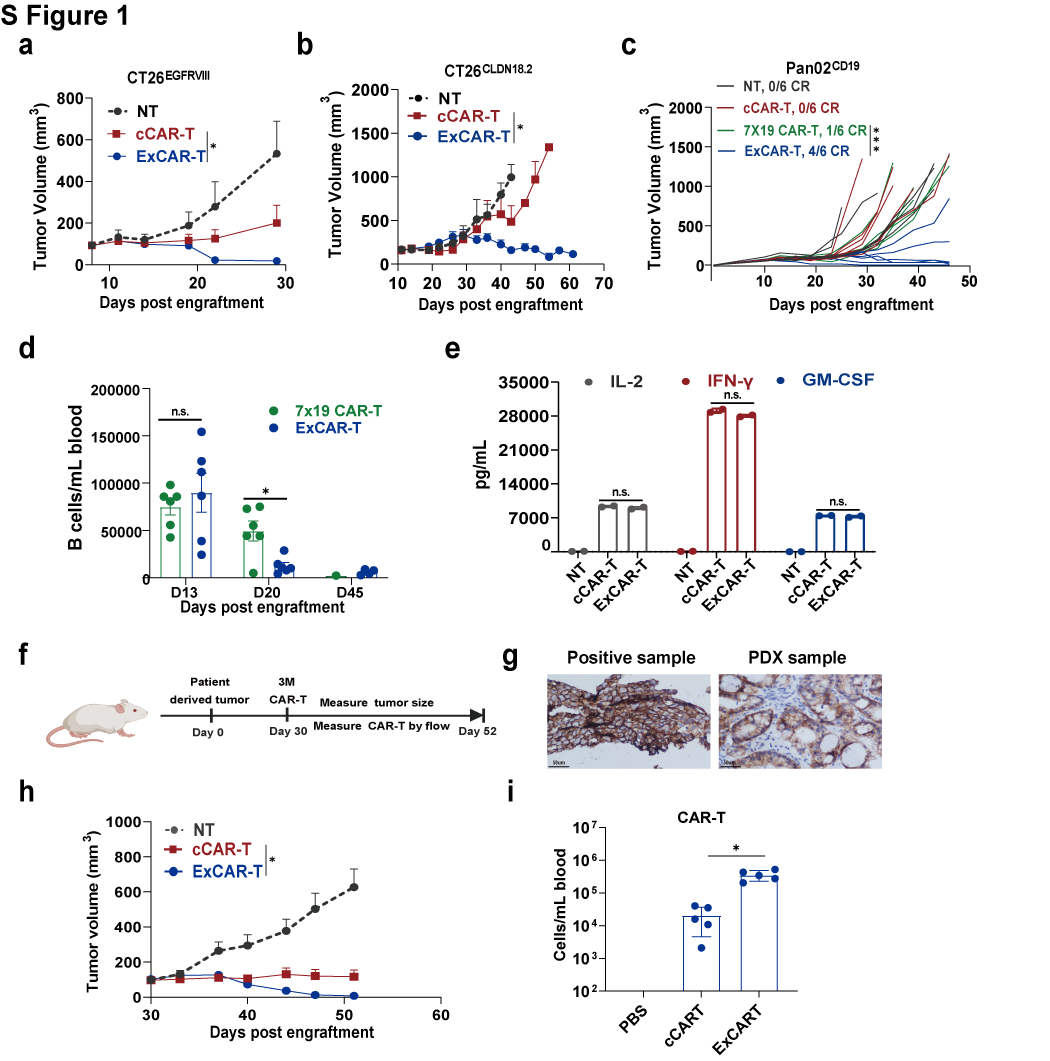
 Supplementary Figure 1. IL-7/XCL1 armored CAR-T cells exhibited improved antitumor effects. a, BALB/c mice were inoculated subcutaneously with CT26^EGFRVIII^ cells and treated with CPA and anti-EGFRVIII CAR-T cells. The tumor volumes are shown (n=8). b, BALB/c mice were inoculated subcutaneously with CT26^CLDN18.2^ cells and treated with cyclophosphamide (CPA) and anti-CLDN18.2 CAR-T cells. The tumor volumes are shown (n=6). c, C57BL/6 mice were inoculated subcutaneously with Pan02^CD19^ cells and treated with CPA and anti-CD19 CAR-T cells. The tumor volume and survival time are shown (n=6). d, B cell counts in the peripheral blood (Day 13 and 20: n = 6; Day 45: n = 1 for 7×19 CAR-T cells, n = 4 for ExCAR-T cells). e, Antigen-specific IL-2, IFN-γ and GM-CSF secretion by CAR-T cells using NUGC4^CLDN18.2^ cells as a target. Target cells (2×10^5^/well) were mixed with anti-hCLDN18.2 cCAR-T cells or ExCAR-T cells (2×10^5^/well). The concentrations of cytokines in the supernatants after 16 hours of coculture were measured via ELISA (n=2). f-g, Experimental design of the hCLDN18.2^+^ PDX model. In the preliminary stage, immunohistochemistry (IHC) was used to screen for hClaudin18.2-positive gastric cancer PDX samples. A positive sample (ID: B00384, tumor block information: B00384-P3-270#) was selected. The gastric cancer PDX sample was thawed and subcutaneously implanted into five NCG mice. When the tumor volume reached 300 mm³, the tumors were expanded by further subcutaneous passaging. Tumor block transplantation was performed on the right upper limb. On day 30 post-implantation,15 tumor-bearing mice were randomly divided into three groups (n=5) based on tumor volume and treated with anti-CLDN18.2 CAR-T cells. h, Tumor growth curves of the different treatment groups (n=5). i, CAR-T-cell count detected by flow cytometry in the CLDN18.2^+^ PDX model on 12 after CAR-T cell treatment (n=5). *P*-values from a-c were performed using two-way ANOVA. *P*-values from d-e and i were determined using two-sided unpaired t test. The data are presented as the mean ± SEM. *****P*<0.0001; ****P*<0.0005; ***P*<0.001; **P*<0.05; n.s., not significant.


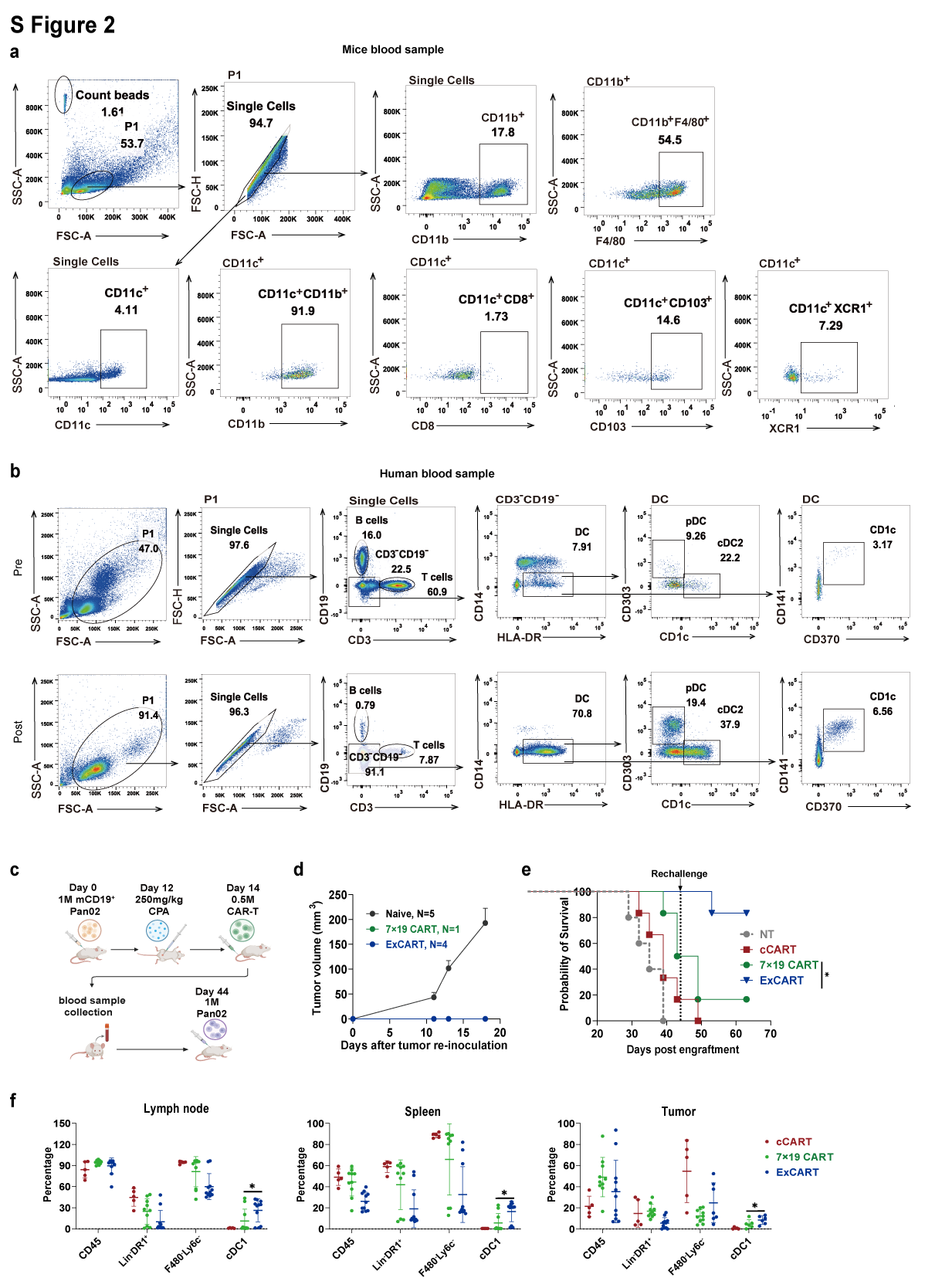


**Supplementary Figure 2. IL-7/XCL1 armored CAR-T cells activate endogenous immune cells. a,** Gating strategy for different mouse DC subsets in the *in vivo* mouse model. **b,** Gating strategy for different human DC subsets in the *in vitro* transwell assay. **c,** Schematic experiment design of Pan02^CD19^ mouse model. **d,** In the Pan02^CD19^ mouse model, D44 C57BL/6 mice were reinoculated subcutaneously (s.c.) with Pan02 cells, and the rechallenge tumor volume is shown (n = 1 for 7×19 CAR-T cells, n = 4 for ExCAR-T cells). **e,** Kaplan‒Meier survival curves for each treatment group; statistical analysis was performed using the log-rank test (n=6). **f,** Percentages of monocytes in lymph node, spleen and tumor samples from the Pan02^CD19^ cell rechallenge mouse model were examined by flow cytometry (n = 5 for cCAR-T cells; n = 10 for 7×19 CAR-T cells and ExCAR-T cells). The data are presented as the mean ± SEM. *P*-values from f were determined using two-sided unpaired t test. *****P*<0.0001; **P*<0.05.


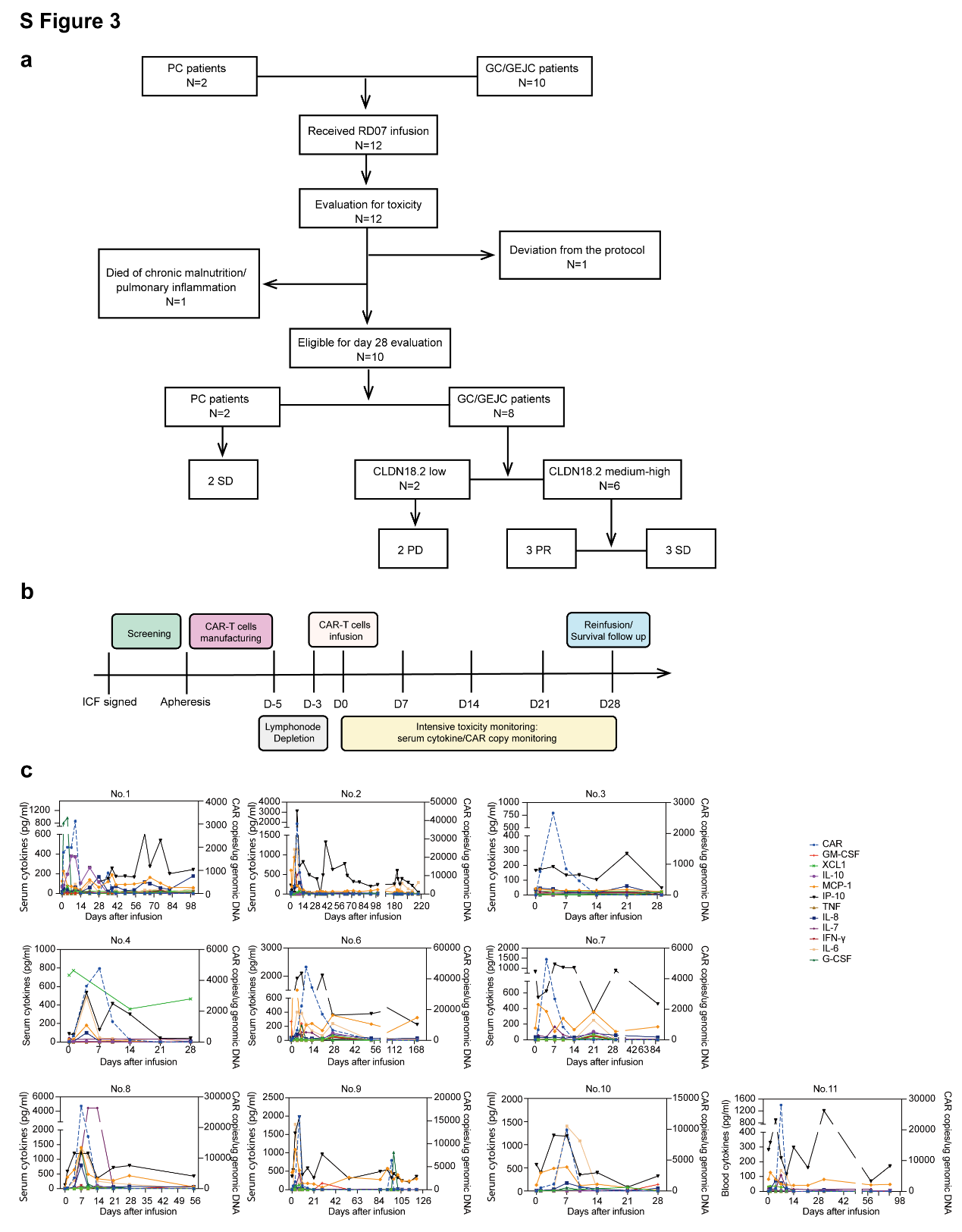


**Supplementary Figure 3. Consort diagram of the RD07 clinical trial, treatment protocol, cellular pharmacokinetics and cytokine levels in peripheral blood of patient. a,** Overview of patient enrollment, treatment and initial efficacy evaluation. **b,** Schematic representation of the clinical trial design. **c,** *In vivo* expansion of CAR-T cells and changes in cytokine levels in the serum.


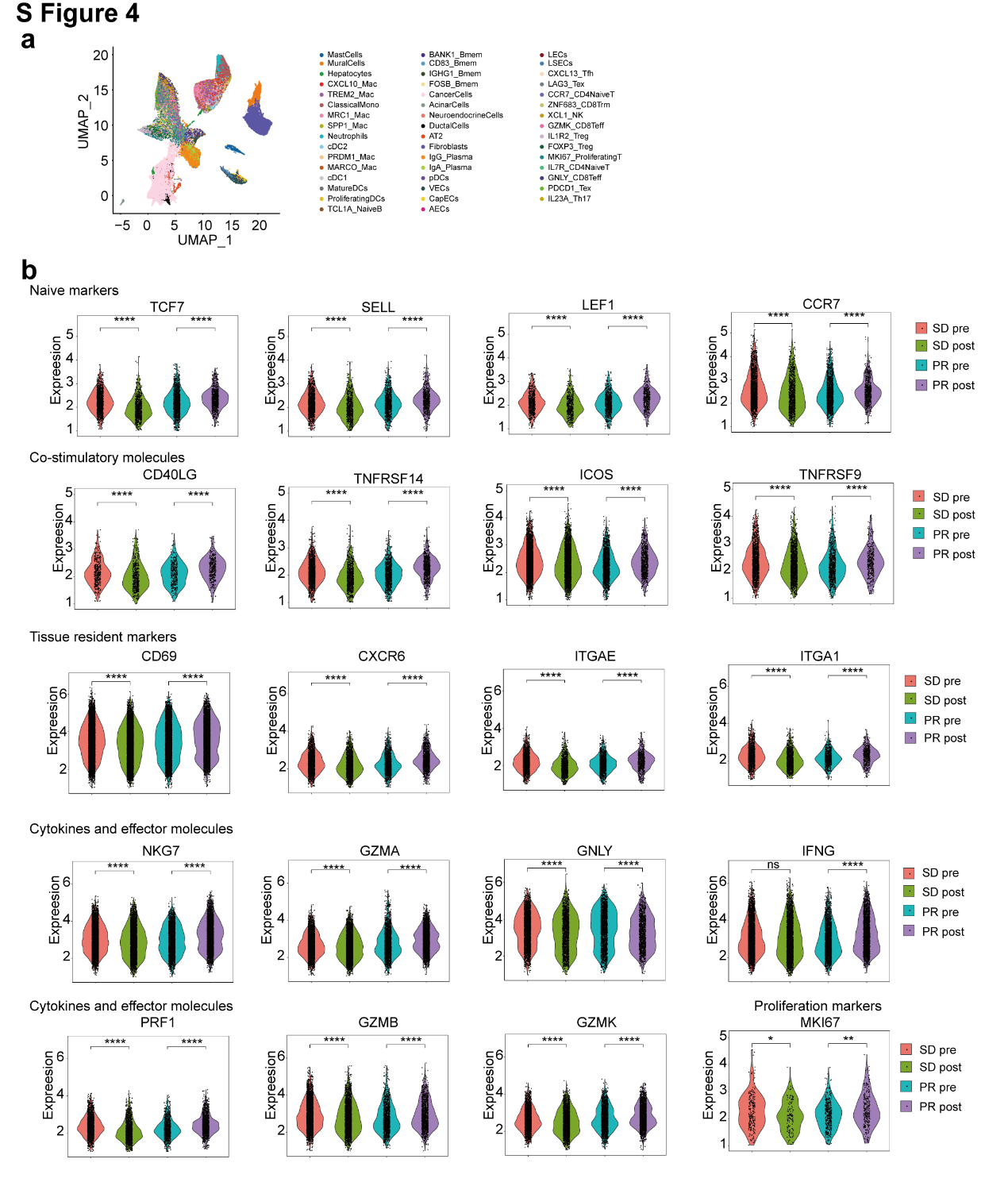


**Supplementary Figure 4. scRNA-seq analysis of the TME in GC patients treated with RD07. a,** UMAP presenting 47 unique cell states color-coded by their corresponding cell lineage or subtype. Each dot in UMAP represents a single cell. **b,** Violin plot illustrating the expression levels of T-cell function-associated genes, including naïve markers (*TCF7*, *SELL*, *LEF1* and *CCR7*), co-stimulatory molecules (*CD40LG*, *TNFRSF14*, *ICOS* and *TNFRSF9*), tissue resident markers (*CD69*, *CXCR6*, *ITGAE* and *ITGA1*), cytokine/effector markers (*NKG7, GZMA, GNLY, IFNG, PRF1, GZMB* and *GZMK*) and a proliferation marker (*MKI67*), in tumor-infiltrated T cells from patients with SD and PR before and after RD07 infusion. Statistical analysis was performed via unpaired two-tailed Wilcoxon–Mann–Whitney U tests. *****P*<0.0001; ***P*<0.001; **P*<0.05; ns, nonsignificant.


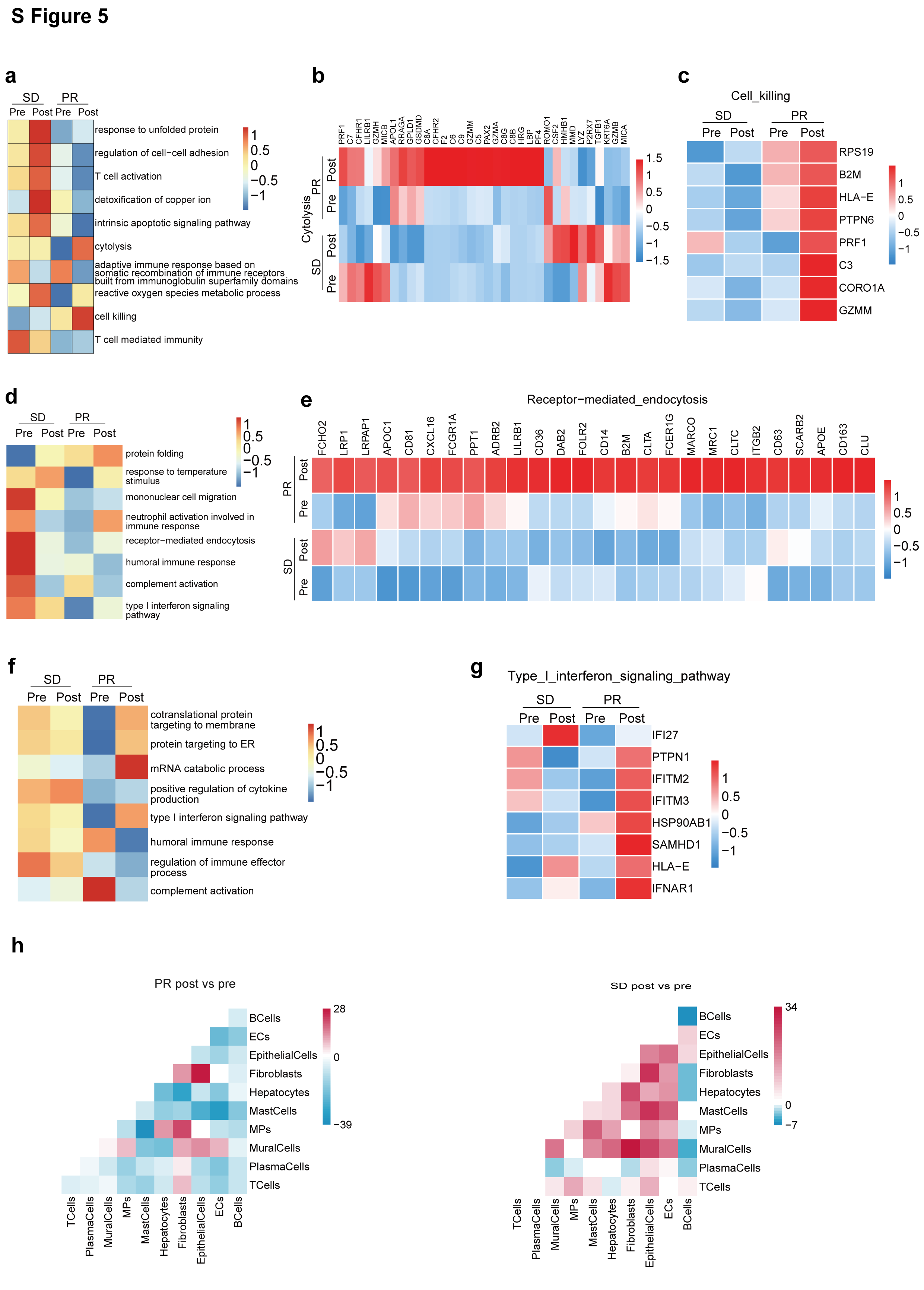


**Supplementary Figure 5.** **Pathway and cellular interaction analysis in GC patients pre- and post-treatment.** **a-c,** GSVA results analysis showing enrichment of pathways such as cytolysis and cell killing in the T cells of SD and PR patient TMEs. Heatmap showing the comparison of gene expression associated with these two pathways. **d-e,** GSVA revealed that the receptor-mediated endocytosis was enriched in MP cells in SD and PR subgroups before and after RD07 treatment. Heatmap showing expression changes in genes associated with endocytosis. **f-g,** GSVA analysis showed that type I interferon signaling pathway was enriched in cDC1s, and the changes in this pathway associated genes in SD and PR patients are shown in the heatmap. The mean GSVA scores were z-score transformed. **h,** The heatmap shows the number of differential interaction pairs between post-treatment and pre-treatment conditions in the SD and PR groups, where red indicates more interaction pairs after treatment and blue indicates fewer interaction pairs.


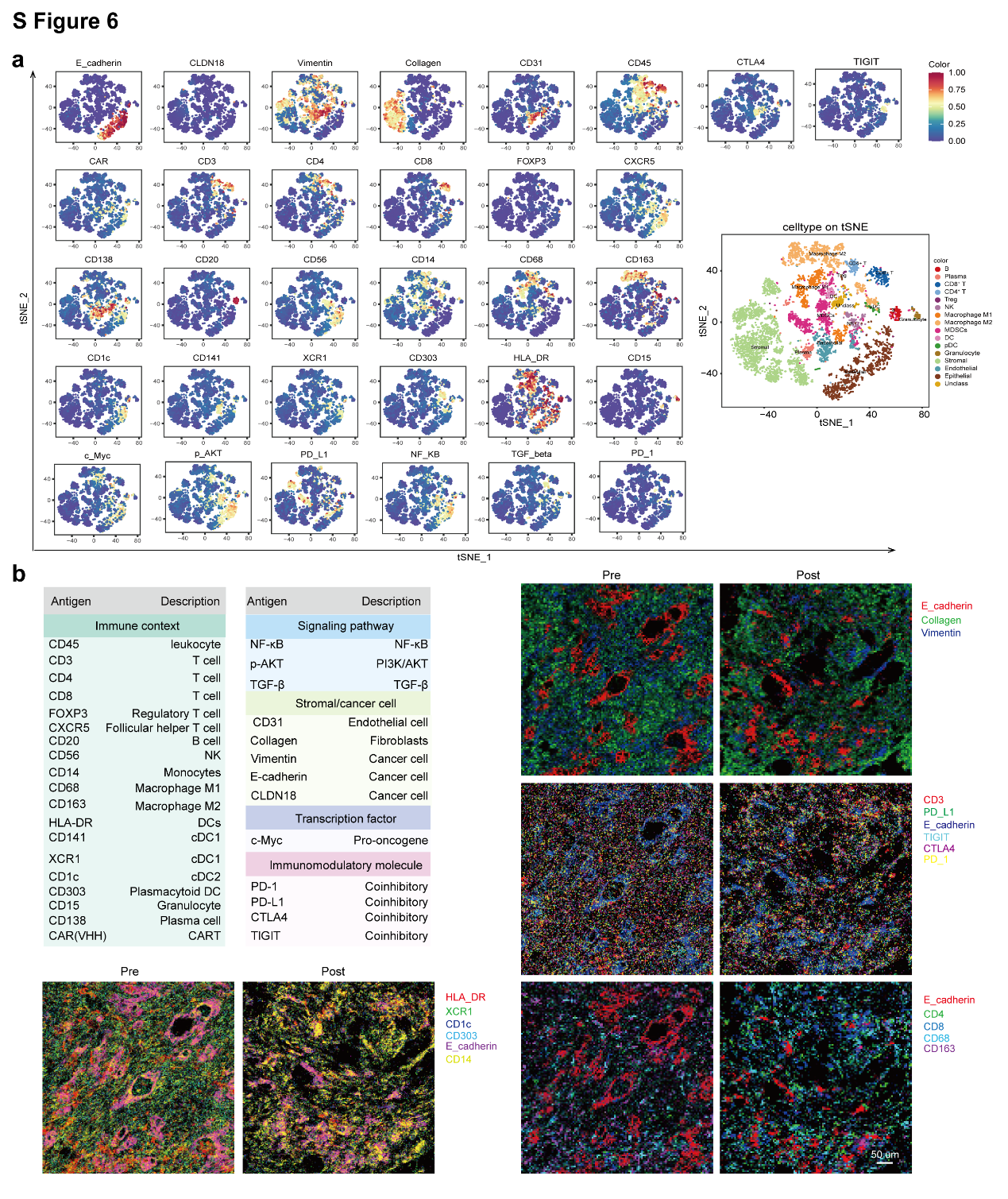


**Supplementary Figure 6. Antibody panel and representative pseudocolored images of marker expression.** **a,** t-SNE visualization of cell types, illustrating the distribution of single cells in slices. Single markers highlighted the distribution of 7100 cells. **b,** A 32-plex antibody panel was used to stain the cancer tissue before and after treatment, and representative IMC images are shown. Scale bars, 50 μm.


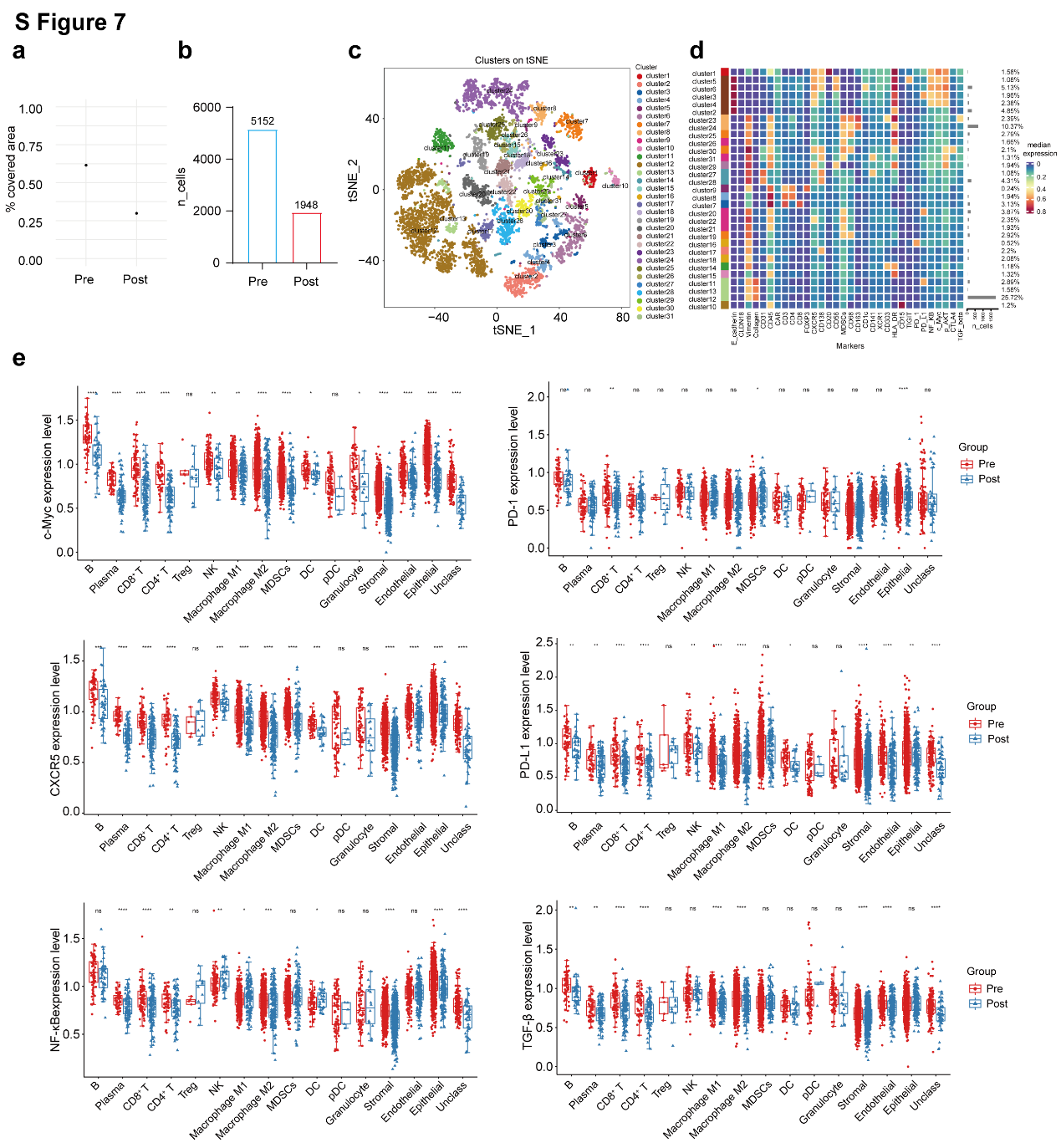


**Supplementary Figure 7. The expression of different proteins in each cell type in the pre- and post-treatment samples.** **a,** The coverage of the signal on the slices pre- and post-treatment. **b,** Number of single cells in a 1 mm^2^ slice. The number of cells decreased from 5152 in the pretreatment sample to 1948 in the posttreatment sample. Thus, a total of 7100 cells were available for analysis**. c,** t-SNE of 31 clusters (details in Supplementary Table 5) highlighting the distribution of 7100 cells. **d,** Expression of markers characteristic of the 31 clusters and ratios among the clusters. **e,** Differential protein expression of immune cells across two slices. Statistical analysis was performed via unpaired two-tailed Wilcoxon–Mann–Whitney U tests. *****P*<0.0001; ****P*<0.0005; ***P*<0.001; **P*<0.05; ns, nonsignificant.

**Supplementary Table 1. Aggregate baseline characteristics of all patients.**

| **Baseline characteristic** | **All patients (*n* =12)** |
| --- | --- |
| Age, years, median (range) | 56.5 (42-70) |
| Male sex, *n* (%) | 4 (33.3) |
| Primary tumor site, *n* (%) |  |
| GC | 9 (75) |
| GEJC | 1 (8.3) |
| PC | 2 (16.7) |
| Time since diagnosis, months, median (range) | 15 (11-26) |
| Extent of disease, *n* (%) |  |
| Locally advanced | 0 (0) |
| Metastatic | 12 (100) |
| Number of metastatic organs, *n* (%) |  |
| ≤2 | 6 (50) |
| ≥3 | 6 (50) |
| Numbers of metastatic organ, median (range) | 2.5 (1-4) |
| Organs involved, primary tumor excluded, *n* (%) |  |
| Liver | 9 (75) |
| Lung | 3 (25) |
| Bone | 4 (33.3) |
| Peritoneum | 1 (8.3) |
| Lymph nodes | 7 (58.3) |
| Other | 7 (58.3) |
| Prior surgical treatment, *n* (%) | 8 (66.7) |
| ECOG performance status score, *n* (%) |  |
| 0 | 0 (0) |
| 1 | 6 (50) |
| 2 | 6 (50) |
| Bridging therapy, *n* (%) | 0 (0) |
| CLDN18.2 expression, *n* (%) ^a^ |  |
| Low expression | 4 (33.3) |
| Medium expression | 4 (33.3) |
| High expression | 4 (33.3) |
| Number of previous systemic regimens, *n* (%) |  |
| 1 | 0 (0) |
| 2 | 6 (50) |
| ≥3 | 6 (50) |
| Prior systemic therapies, *n* (%) |  |
| Chemotherapy | 12 (100) |
| Anti-PD-1/PD-L1 antibody | 11 (91.7) |
| Tyrosine kinase inhibitor^b^ | 8 (66.7) |
| **Baseline characteristic** | **GC/GEJC patient (n=10)** |
| Histological subtype (Lauren classification), *n* (%) |  |
| Intestinal | 2 (20) |
| Diffuse | 3 (30) |
| Mixed | 2 (20) |
| Unknown | 3 (30) |
| Histological (WHO) classification, *n* (%) |  |
| Mucinous adenocarcinoma | 10 (100) |
| Signet-ring cell carcinoma | 0 (0) |
| HER2 status, *n* (%) |  |
| Positive | 0 (0) |
| Negative | 10 (100) |

^a^The expression level of CLDN18.2 was assessed using immunohistochemical staining, with a grading system of 0 to 3+ based on staining intensity, which was then multiplied by the percentage of positively stained tumor cells. A low expression level was defined as any staining intensity with a positivity percentage of less than 40% or an intensity score of 1+ with any positivity percentage. A medium expression level was defined as an intensity score of 2+ or 3+, with a positivity percentage between 40% (inclusive) and 69%. A high expression level was defined as an intensity score of 2+ or 3+, with a positivity percentage of 70% or higher. ^b^Tyrosine kinase inhibitors: apatinib, anlotinib, etc.

**Supplementary Table 2. Patients' baseline data, treatment regimens and best responses.**

| **Patient No.** | **Age** | **Sex** | **Diagnosis** | **Prior lines** | **Claudin18.2 expression** | **Preconditioning regimen** | **Cycles of infusion** | **Dose(s) of each infusion (10^7^/kg)** | **First efficacy evaluation** | **Response to**  **reinfusions** | **Best overall response** |
| --- | --- | --- | --- | --- | --- | --- | --- | --- | --- | --- | --- |
| 1 | 56 | F | PC | 2 | 2+, 10% 1+, 20% | CTX: 225 mg/m^2^×3 days  Nab-Pax: 100 mg/m^2^×3 days（only used in C1,C3 and C4） | 4 | 0.1,  0.5,  1,  2 | **SD** | **SD/SD** | **SD** |
| 2 | 57 | M | GC | 3 | 2~3+, 80% | Flu:25 mg/m^2^×2days CTX:500 mg/m^2^×2days（only used in C1 and C5） | 5 | 0.5,  0.5,  1,  1,  1 | **PR** | **PR/PR/PR** | **PR** |
| 3 | 60 | M | GC | 3 | 2+, 30% | Flu: 25 mg/m^2^×3 days CTX: 300 mg/m^2^×3 days | 1 | 0.79 | **PD** | **NA** | **PD** |
| 4 | 60 | F | GC | 2 | 1+, 30% | Flu: 25 mg/m^2^×2 days CTX: 500 mg/m^2^×2 days | 1 | 2 | **PD** | **NA** | **PD** |
| 5 | 47 | F | GC | 2 | 1+, 30% | Flu: 25 mg/m^2^×2 days CTX: 500 mg/m^2^×2 days | 1 | 2 | **NA** | **NA** | **NA** |
| 6 | 45 | F | GC | 3 | 2+, 50% | Flu: 25 mg/m^2^×3 days CTX: 300 mg/m^2^×3 days | 1 | 0.5 | **SD** | **NA** | **SD** |
| 7 | 70 | F | GC | 2 | 3+, 20% 2+, 25% 1+, 10% | Flu: 25 mg/m^2^×2 days CTX: 500 mg/m^2^×2 days | 1 | 0.5 | **SD** | **NA** | **SD** |
| 8 | 64 | F | GEJC | 3 | 3+, 30% 2+, 20% 1+, 10% | Flu: 25 mg/m^2^×2 days CTX: 500 mg/m^2^×2 days | 1 | 0.5 | **PR** | **NA** | **SD** |
| 9 | 42 | F | GC | 2 | 3+, 70% 2+, 15% 1+, 5% | Flu: 25 mg/m^2^×2 days CTX: 300 mg/m^2^×2 days Nab-Pax: 100 mg/m^2^×1 day | 2 | 0.75,  1 | **SD** | **SD** | **SD** |
| 10 | 51 | M | PC | 3 | 3+, 60% | Flu: 25 mg/m^2^×2 days CTX: 250 mg/m^2^×3 days Nab-Pax: 100 mg/m^2^×1 day | 1 | 0.75 | **SD** | **NA** | **SD** |
| 11 | 54 | F | GC | 1 | 3+, 70% 2+, 10% 1+, 10% | Flu: 25 mg/m^2^×2 days CTX: 250 mg/m^2^×3 days Nab-Pax: 100 mg×1 day | 1 | 0.75 | **PR** | **NA** | **PR** |
| 12 | 57 | M | GC | 3 | 3+, 70% 2+, 20% 1+, 5% | Flu: 25 mg/m^2^×2 days CTX: 250 mg/m^2^×3 days Nab-Pax: 100 mg×1 day | 2 | 1.5,  2 | **NA** | **NA** | **NA** |

Abbreviations: CTX, cyclophosphamide; Flu, fludarabine; Nab-Pax, nab-paclitaxel; CR, complete response; PR, partial response; SD, stable disease; PD, progressive disease; NA, not applicable; first efficacy evaluation, tumor responses were evaluated at 28 days after CAR-T-cell infusion; response to reinfusions, patients received multiple infusions underwent multiple efficacy evaluations during treatment; best overall response, the best confirmed response achieved by the patient during treatment.

| **Patient No.** | **Puncture site** | **First efficacy evaluation** |  |
| --- | --- | --- | --- |
| 6 | Pelvic lymph nodes | SD |  |
| 7 | Liver | SD |  |
| 8 | Retroperitoneal lymph nodes | PR |  |
| 9 | | Ovary | SD |
| 11 | | Liver | PR |

**Supplementary Table 3. Information on tumor tissues obtained from patients with GC for scRNA-seq.**

| **Supplementary Table 4. The mutation of IL7R in patients with different outcomes.** | | | | |  |
| --- | --- | --- | --- | --- | --- |
| **Patient No./**  **Response** | **Position** | **Consequence type** | **Feature ID** | **HGVS.c** | |
| 8, 11/PR | Chr5: 35857105 | intron_variant; intron_variant; intron_variant; intron_variant; intron_variant; intron_variant; intron_variant | ENST00000303115.8; ENST00000514217.5; ENST00000506850.5; ENST00000511982.1; ENST00000511031.1; ENST00000508941.5; ENST00000515665.1 | c.82+46C>T; n.82+46C>T; c.82+46C>T; c.82+46C>T; n.217-3747C>T; c.82+46C>T; c.82+46C>T | |
| 6/SD | Chr5: 35871088 | missense_variant; missense_variant; upstream_gene_variant; upstream_gene_variant; upstream_gene_variant; downstream_gene_variant; downstream_gene_variant; non_coding_transcript_exon_variant | ENST00000303115.8; ENST00000506850.5; ENST00000505093.1; ENST00000505875.1; ENST00000509668.1; ENST00000511982.1; ENST00000511031.1; ENST00000514217.5 | c.412G>A; c.412G>A; c.-2446G>A; n.-4326G>A; n.-2113G>A; c.*3551G>A; n.*3623G>A; n.412G>A | |
|  | Chr5: 35876347 | missense_variant; 3_prime_UTR_variant; 3_prime_UTR_variant; downstream_gene_variant; downstream_gene_variant; non_coding_transcript_exon_variant; non_coding_transcript_exon_variant | ENST00000303115.8; ENST00000514217.5; ENST00000505093.1; ENST00000506850.5; ENST00000509668.1; ENST00000514217.5; ENST00000505875.1 | c.1241C>T; n.*435C>T; c.*358C>T; c.*756C>T; n.*826C>T; n.*435C>T; n.539C>T | |
|  | Chr5: 35857075 | intron_variant; intron_variant; intron_variant; intron_variant; intron_variant; intron_variant; intron_variant | ENST00000303115.8; ENST00000514217.5; ENST00000506850.5; ENST00000511982.1; ENST00000511031.1; ENST00000508941.5; ENST00000515665.1 | c.82+16G>C; n.82+16G>C; c.82+16G>C; c.82+16G>C; n.217-3777G>C; c.82+16G>C; c.82+16G>C | |
|  | Chr5: 35857133 | intron_variant; intron_variant; intron_variant; intron_variant; intron_variant; intron_variant; intron_variant | ENST00000303115.8; ENST00000514217.5; ENST00000506850.5; ENST00000511982.1; ENST00000511031.1; ENST00000508941.5; ENST00000515665.1 | c.82+74C>G; n.82+74C>G; c.82+74C>G; c.82+74C>G; n.217-3719C>G; c.82+74C>G; c.82+74C>G | |
|  | Chr5: 35857160 | intron_variant; intron_variant; intron_variant; intron_variant; intron_variant; intron_variant; intron_variant | ENST00000303115.8; ENST00000514217.5; ENST00000506850.5; ENST00000511982.1; ENST00000511031.1; ENST00000508941.5; ENST00000515665.1 | c.82+101A>G; n.82+101A>G; c.82+101A>G; c.82+101A>G; n.217-3692A>G; c.82+101A>G; c.82+101A>G | |
|  | Chr5: 35860966 | missense_variant; missense_variant; missense_variant; downstream_gene_variant; downstream_gene_variant; non_coding_transcript_exon_variant; non_coding_transcript_exon_variant | ENST00000303115.8; ENST00000506850.5; ENST00000511982.1; ENST00000508941.5; ENST00000515665.1; ENST00000514217.5; ENST00000511031.1 | c.197T>C; c.197T>C; c.197T>C; c.*18T>C; c.*40T>C; n.197T>C; n.331T>C | |
|  | Chr5: 35861057 | downstream_gene_variant; downstream_gene_variant; intron_variant; intron_variant; intron_variant; intron_variant; intron_variant | ENST00000508941.5; ENST00000515665.1; ENST00000303115.8; ENST00000514217.5; ENST00000506850.5; ENST00000511982.1; ENST00000511031.1 | c.*109A>G; c.*131A>G; c.221+67A>G; n.221+67A>G; c.221+67A>G; c.221+67A>G; n.355+67A>G | |
|  | Chr5: 35861166 | downstream_gene_variant; downstream_gene_variant; intron_variant; intron_variant; intron_variant; intron_variant; intron_variant | ENST00000508941.5; ENST00000515665.1; ENST00000303115.8; ENST00000514217.5; ENST00000506850.5; ENST00000511982.1; ENST00000511031.1 | c.*218T>G; c.*240T>G; c.221+176T>G; n.221+176T>G; c.221+176T>G; c.221+176T>G; n.355+176T>G | |
|  | Chr5: 35867102 | intron_variant; intron_variant; intron_variant; intron_variant; intron_variant | ENST00000303115.8; ENST00000514217.5; ENST00000506850.5; ENST00000511982.1; ENST00000511031.1 | c.222-204A>T; n.222-204A>T; c.222-204A>T; c.222-204A>T; n.356-204A>T | |
|  | Chr5: 35871171 | synonymous_variant; synonymous_variant; upstream_gene_variant; upstream_gene_variant; upstream_gene_variant; downstream_gene_variant; downstream_gene_variant; non_coding_transcript_exon_variant | ENST00000303115.8; ENST00000506850.5; ENST00000505093.1; ENST00000505875.1; ENST00000509668.1; ENST00000511982.1; ENST00000511031.1; ENST00000514217.5 | c.495C>T; c.495C>T; c.-2363C>T; n.-4243C>T; n.-2030C>T; c.*3634C>T; n.*3706C>T; n.495C>T | |
| 7/SD | Chr5: 35871088 | missense_variant; missense_variant; upstream_gene_variant; upstream_gene_variant; upstream_gene_variant; downstream_gene_variant; downstream_gene_variant; non_coding_transcript_exon_variant | ENST00000303115.8; ENST00000506850.5; ENST00000505093.1; ENST00000505875.1; ENST00000509668.1; ENST00000511982.1; ENST00000511031.1; ENST00000514217.5 | c.412G>A; c.412G>A; c.-2446G>A; n.-4326G>A; n.-2113G>A; c.*3551G>A; n.*3623G>A; n.412G>A | |
|  | Chr5: 35857075 | intron_variant; intron_variant; intron_variant; intron_variant; intron_variant; intron_variant; intron_variant | ENST00000303115.8; ENST00000514217.5; ENST00000506850.5; ENST00000511982.1; ENST00000511031.1; ENST00000508941.5; ENST00000515665.1 | c.82+16G>C; n.82+16G>C; c.82+16G>C; c.82+16G>C; n.217-3777G>C; c.82+16G>C; c.82+16G>C | |
|  | Chr5: 35857133 | intron_variant; intron_variant; intron_variant; intron_variant; intron_variant; intron_variant; intron_variant | ENST00000303115.8; ENST00000514217.5; ENST00000506850.5; ENST00000511982.1; ENST00000511031.1; ENST00000508941.5; ENST00000515665.1 | c.82+74C>G; n.82+74C>G; c.82+74C>G; c.82+74C>G; n.217-3719C>G; c.82+74C>G; c.82+74C>G | |
|  | Chr5: 35857160 | intron_variant; intron_variant; intron_variant; intron_variant; intron_variant; intron_variant; intron_variant | ENST00000303115.8; ENST00000514217.5; ENST00000506850.5; ENST00000511982.1; ENST00000511031.1; ENST00000508941.5; ENST00000515665.1 | c.82+101A>G; n.82+101A>G; c.82+101A>G; c.82+101A>G; n.217-3692A>G; c.82+101A>G; c.82+101A>G | |
|  | Chr5: 35860966 | missense_variant; missense_variant; missense_variant; downstream_gene_variant; downstream_gene_variant; non_coding_transcript_exon_variant; non_coding_transcript_exon_variant | ENST00000303115.8; ENST00000506850.5; ENST00000511982.1; ENST00000508941.5; ENST00000515665.1; ENST00000514217.5; ENST00000511031.1 | c.197T>C; c.197T>C; c.197T>C; c.*18T>C; c.*40T>C; n.197T>C; n.331T>C | |
|  | Chr5: 35861057 | downstream_gene_variant; downstream_gene_variant; intron_variant; intron_variant; intron_variant; intron_variant; intron_variant | ENST00000508941.5; ENST00000515665.1; ENST00000303115.8; ENST00000514217.5; ENST00000506850.5; ENST00000511982.1; ENST00000511031.1 | c.*109A>G; c.*131A>G; c.221+67A>G; n.221+67A>G; c.221+67A>G; c.221+67A>G; n.355+67A>G | |
|  | Chr5: 35857105 | intron_variant; intron_variant; intron_variant; intron_variant; intron_variant; intron_variant; intron_variant | ENST00000303115.8; ENST00000514217.5; ENST00000506850.5; ENST00000511982.1; ENST00000511031.1; ENST00000508941.5; ENST00000515665.1 | c.82+46C>T; n.82+46C>T; c.82+46C>T; c.82+46C>T; n.217-3747C>T; c.82+46C>T; c.82+46C>T | |
|  | Chr5: 35861166 | downstream_gene_variant; downstream_gene_variant; intron_variant; intron_variant; intron_variant; intron_variant; intron_variant | ENST00000508941.5; ENST00000515665.1; ENST00000303115.8; ENST00000514217.5; ENST00000506850.5; ENST00000511982.1; ENST00000511031.1 | c.*218T>G; c.*240T>G; c.221+176T>G; n.221+176T>G; c.221+176T>G; c.221+176T>G; n.355+176T>G | |
|  | Chr5: 35871171 | synonymous_variant; synonymous_variant; upstream_gene_variant; upstream_gene_variant; upstream_gene_variant; downstream_gene_variant; downstream_gene_variant; non_coding_transcript_exon_variant | ENST00000303115.8; ENST00000506850.5; ENST00000505093.1; ENST00000505875.1; ENST00000509668.1; ENST00000511982.1; ENST00000511031.1; ENST00000514217.5 | c.495C>T; c.495C>T; c.-2363C>T; n.-4243C>T; n.-2030C>T; c.*3634C>T; n.*3706C>T; n.495C>T | |
| 9/SD | Chr5: 35857364 | intron_variant; intron_variant; intron_variant; intron_variant; intron_variant; intron_variant; intron_variant | ENST00000303115.8; ENST00000514217.5; ENST00000506850.5; ENST00000511982.1; ENST00000511031.1; ENST00000508941.5; ENST00000515665.1 | c.82+305T>G; n.82+305T>G; c.82+305T>G; c.82+305T>G; n.217-3488T>G; c.82+305T>G; c.82+305T>G | |
|  | Chr5: 35871088 | missense_variant; missense_variant; upstream_gene_variant; upstream_gene_variant; upstream_gene_variant; downstream_gene_variant; downstream_gene_variant; non_coding_transcript_exon_variant | ENST00000303115.8; ENST00000506850.5; ENST00000505093.1; ENST00000505875.1; ENST00000509668.1; ENST00000511982.1; ENST00000511031.1; ENST00000514217.5 | c.412G>A; c.412G>A; c.-2446G>A; n.-4326G>A; n.-2113G>A; c.*3551G>A; n.*3623G>A; n.412G>A | |
|  | Chr5: 35857075 | intron_variant; intron_variant; intron_variant; intron_variant; intron_variant; intron_variant; intron_variant | ENST00000303115.8; ENST00000514217.5; ENST00000506850.5; ENST00000511982.1; ENST00000511031.1; ENST00000508941.5; ENST00000515665.1 | c.82+16G>C; n.82+16G>C; c.82+16G>C; c.82+16G>C; n.217-3777G>C; c.82+16G>C; c.82+16G>C | |
|  | Chr5: 35857133 | intron_variant; intron_variant; intron_variant; intron_variant; intron_variant; intron_variant; intron_variant | ENST00000303115.8; ENST00000514217.5; ENST00000506850.5; ENST00000511982.1; ENST00000511031.1; ENST00000508941.5; ENST00000515665.1 | c.82+74C>G; n.82+74C>G; c.82+74C>G; c.82+74C>G; n.217-3719C>G; c.82+74C>G; c.82+74C>G | |
|  | Chr5: 35857160 | intron_variant; intron_variant; intron_variant; intron_variant; intron_variant; intron_variant; intron_variant | ENST00000303115.8; ENST00000514217.5; ENST00000506850.5; ENST00000511982.1; ENST00000511031.1; ENST00000508941.5; ENST00000515665.1 | c.82+101A>G; n.82+101A>G; c.82+101A>G; c.82+101A>G; n.217-3692A>G; c.82+101A>G; c.82+101A>G | |
|  | Chr5: 35860966 | missense_variant; missense_variant; missense_variant; downstream_gene_variant; downstream_gene_variant; non_coding_transcript_exon_variant; non_coding_transcript_exon_variant | ENST00000303115.8; ENST00000506850.5; ENST00000511982.1; ENST00000508941.5; ENST00000515665.1; ENST00000514217.5; ENST00000511031.1 | c.197T>C; c.197T>C; c.197T>C; c.*18T>C; c.*40T>C; n.197T>C; n.331T>C | |
|  | chr5: 35875491 | intron_variant; intron_variant; intron_variant; intron_variant; intron_variant; non_coding_transcript_exon_variant | ENST00000303115.8; ENST00000514217.5; ENST00000506850.5; ENST00000505093.1; ENST00000509668.1; ENST00000505875.1 | c.801-21A>T; n.538-21A>T; c.707-21A>T; c.116-21A>T; n.543-21A>T; n.78A>T | |
|  | chr5: 35857105 | intron_variant; intron_variant; intron_variant; intron_variant; intron_variant; intron_variant; intron_variant | ENST00000303115.8; ENST00000514217.5; ENST00000506850.5; ENST00000511982.1; ENST00000511031.1; ENST00000508941.5; ENST00000515665.1 | c.82+46C>T; n.82+46C>T; c.82+46C>T; c.82+46C>T; n.217-3747C>T; c.82+46C>T; c.82+46C>T | |
|  | chr5: 35861050 | downstream_gene_variant; downstream_gene_variant; intron_variant; intron_variant; intron_variant; intron_variant; intron_variant | ENST00000508941.5; ENST00000515665.1; ENST00000303115.8; ENST00000514217.5; ENST00000506850.5; ENST00000511982.1; ENST00000511031.1 | c.*102C>G; c.*124C>G; c.221+60C>G; n.221+60C>G; c.221+60C>G; c.221+60C>G; n.355+60C>G | |
|  | chr5: 35861166 | downstream_gene_variant; downstream_gene_variant; intron_variant; intron_variant; intron_variant; intron_variant; intron_variant | ENST00000508941.5; ENST00000515665.1; ENST00000303115.8; ENST00000514217.5; ENST00000506850.5; ENST00000511982.1; ENST00000511031.1 | c.*218T>G; c.*240T>G; c.221+176T>G; n.221+176T>G; c.221+176T>G; c.221+176T>G; n.355+176T>G | |

**Supplementary Table 5. Cells in the IMC classified into 31 different clusters.**

| **Cluster_ID** | **Marker** | **Cell type** |
| --- | --- | --- |
| cluster1 | Vimentin+ CD45+ CD20+ HLA_DR+ | B |
| cluster2 | E_cadherin+ HLA_DR+ | Epithelial |
| cluster3 | E_cadherin+PD_L1+ HLA_DR+ | Epithelial |
| cluster4 | E_cadherin+ HLA_DR+ | Epithelial |
| cluster5 | E_cadherin+ CD56+ TIGIT+ HLA_DR+ | Epithelial |
| cluster6 | E_cadherin+ CD56+ CD1c+ HLA_DR+ | Epithelial |
| cluster7 | Vimentin+ CD45+ CD3+ CD8+ | CD8+ T |
| cluster8 | Vimentin+ CD45+ CD3+ CD4+ | CD4+ T |
| cluster9 | Vimentin+ CD45+ CD4+ FOXP3+ | Treg |
| cluster10 | Vimentin+ CD45+ CD15+ | Granulocyte |
| cluster11 | Vimentin+ Collagen+ PD_L1+ | Stromal |
| cluster12 | Vimentin+ Collagen+ | Stromal |
| cluster13 | Vimentin+ Collagen+ | Stromal |
| cluster14 | Vimentin+ CD45(low)+ CD14- CD303+ HLA_DR+ | pDC |
| cluster15 | Vimentin+ CD45+ CD14- HLA_DR+ | DC |
| cluster16 | Vimentin+ PD1+ | Unclass |
| cluster17 | Vimentin+ CD45- CD138+ | Plasma |
| cluster18 | Vimentin+ CD45+ | Unclass |
| cluster19 | Vimentin+ CD45+ CD4(low) CD14+ CD68+ HLA_DR (low) | Macrophage M1 |
| cluster20 | Vimentin+ CD45+ CD14+ PD_L1+ HLA_DR- | MDSCs |
| cluster21 | Vimentin+ CD45+ CD14+ HLA_DR+ | MDSCs |
| cluster22 | Vimentin+ CD45+ CD14+ HLA_DR+ | MDSCs |
| cluster23 | Vimentin+ CD45+ CD4+ CD14+ CD68+ CD163+ HLA_DR+ CD303+ | Macrophage M2 |
| cluster24 | Vimentin+ CD45+ CD4(low) CD14+ CD68+ CD163+ HLA_DR+ | Macrophage M2 |
| cluster25 | Vimentin+ CD45+ CD4(low) CD14+ CD68+ HLA_DR+ | Macrophage M1 |
| cluster26 | Vimentin+ CD45+ CD14+ HLA_DR+ | MDSCs |
| cluster27 | Vimentin+ CD31+ CD138+ CD141+ | Endothelial |
| cluster28 | Vimentin+ CD31+ CD138+ | Endothelial |
| cluster29 | Vimentin+ CD45+ CD56+ HLA_DR+ | NK |
| cluster30 | Vimentin+ CD45+ CD14+ CD68+ HLA_DR+ CTLA4+ | Macrophage M1 |
| cluster31 | Vimentin+ CD45+ CD14+ CD141+ HLA_DR- | MDSCs |

**Materials and methods**

**Preparation of mouse CAR-T cells**

The Claudin18.2 VHH nanobody was generated by Nanjing Bioheng Biotech Co., Ltd. VHH and scFv sequences were linked to the mouse CD8α transmembrane domain and the human 4-1BB plus mouse CD3ζ intracellular domains to construct a second-generation CAR, which was cloned into the MSCV vector. To enable co-expression of murine IL-7 and XCL1 alongside the CAR, the three coding sequences were separated by a 2A peptide. All constructs were synthesized by General Biol. CAR plasmid and pCL-Eco were introduced into 293T packaging cells using Lipofectamine Reagent (Thermo Fisher Scientific). 72h later, the culture supernatants were collected for subsequent use. Mouse T cells were activated for 2 days with anti-CD3 (BioLegend, 100340, 1 µg/mL), anti-CD28 (BioLegend, 102121, 1 µg/mL), and IL-2 (50 IU; Jiangsu Sihuan Bioengineering Co., Ltd.), and then cultured with the harvested supernatants on RetroNectin-coated plates (Takara Bio). The cells were further cultured for an additional 3 days with IL-2 (50 IU) for subsequent experiments.

***In vivo* evaluation of antitumor activity of murine CAR-T cells**

A syngeneic CLDN18.2 tumor model was established by implanting Pan02 cells expressing human CLDN18.2 (1×10⁶ cells per mouse) into the right flank of C57BL/6 mice. Following tumor establishment, cyclophosphamide was administered intraperitoneally at a dose of 250 mg/kg on day 9. Three days later (day 12), 5×10⁵ murine CLDN18.2 CAR-T cells generated from donor mice were administered via intravenous injection (i.v.). For tumor rechallenge, mice that achieved complete regression following ExCAR-T therapy were re-inoculated subcutaneously with Pan02 cells on the left flank. An EGFRvIII-expressing syngeneic tumor model was generated by subcutaneous implantation of CT26EGFRvIII cells (1 × 10⁶ cells per mouse) into the right flank of BALB/c mice. Lymphodepletion was achieved by intraperitoneal administration of cyclophosphamide (200 mg/kg) on day 7 after tumor implantation. Mice subsequently received an intravenous infusion of anti-EGFRvIII CAR-T cells (1 × 10⁶ cells) on day 10.

For the Pan02-CD19 model, C57BL/6 mice were implanted subcutaneously with Pan02 cells expressing murine CD19 at a dose of 5×10⁵ cells per animal. Cyclophosphamide (200 mg/kg) was administered intraperitoneally on day 10, followed by intravenous delivery of murine CD19-specific CAR-T cells (2×10⁶ cells) on day 13.

Tumor progression was assessed twice per week using digital caliper measurements, and survival was recorded throughout the study period. In selected experiments, peripheral blood samples were collected to quantify circulating CAR-T cells.

**Quality control, dimension reduction and clustering**

Data processing, dimensionality reduction, and clustering were conducted using Scanpy (v1.8.1) in Python (v3.7). Genes detected in fewer than 5 cells were excluded, and low-quality cells were removed based on the following criteria: <200 detected genes, extremely high gene counts (top 2%), high UMI counts (top 2%), or mitochondrial gene content >20%. The raw count matrix was normalized to total counts per cell and log-transformed. The top 2000 highly variable genes (flavor = “seurat”) were selected for downstream analyses, followed by PCA on the scaled matrix. The top 20 principal components were used for clustering and visualization. Cell-type annotation was performed using Cell-ID, and cell–cell interaction analysis was conducted with CellPhoneDB.

**Differentially expressed genes (DEGs) and pathway enrichment analysis**

DEGs were identified using scanpy.tl.rank_genes_groups with the Wilcoxon rank-sum test under default settings. Genes were considered as DEGs if they were expressed in >10% of cells in either group and showed an average log2 fold change >1. P values were adjusted using the Benjamini–Hochberg method, and an adjusted p value <0.05 was regarded as statistically significant. Functional enrichment analyses, including Gene Ontology (GO) and Kyoto Encyclopedia of Genes and Genomes (KEGG), were conducted using the R package clusterProfiler (v3.16.1), with pathways of adjusted p value <0.05 considered significantly enriched.

Gene set enrichment analysis (GSEA) was performed using DEGs from each cluster compared with those from the SD and PR groups. For pathway activity at the single-cell level, GSVA was implemented using scGSVA (ssGSEA-based), which calculates enrichment scores for individual cells and generates pathway activity matrices. Differential pathway enrichment scores were further evaluated using the limma package, and pathways with |t| > 1.96 were considered significantly different among cell types.

**Whole-exome sequencing**

The PBMCs of patients before RD07 cell therapy were collected, and genomic DNA (gDNA) was extracted. The fragmented gDNA was selected and subjected to standard MGI Genome Analyzer library preparation according to the manufacturer’s protocol. High-quality reads were mapped to the human reference genome (GRCh38) using Burrows–Wheeler Aligner (BWA-v0.7.15). All the variants were further manually reviewed by visual inspection. Sequencing and data analysis were conducted at BGI (Wuhan, China).
